# Oct4 confers stemness and radioresistance to head and neck squamous cell carcinoma by regulating the homologous recombination factors PSMC3IP and RAD54L

**DOI:** 10.1038/s41388-021-01842-1

**Published:** 2021-06-02

**Authors:** Jacqueline Nathansen, Vasyl Lukiyanchuk, Linda Hein, Maya-Isabel Stolte, Kerstin Borgmann, Steffen Löck, Ina Kurth, Michael Baumann, Mechthild Krause, Annett Linge, Anna Dubrovska

**Affiliations:** 1 OncoRay–National Center for Radiation Research in Oncology, Faculty of Medicine and University Hospital Carl Gustav Carus, Technische Universität Dresden, Helmholtz-Zentrum Dresden-Rossendorf, Dresden, Germany; 2 Helmholtz-Zentrum Dresden-Rossendorf, Institute of Radiooncology-OncoRay, Dresden, Germany; 3 Laboratory of Radiobiology and Experimental Radiooncology, Center of Oncology, University Medical Center Hamburg-Eppendorf, Hamburg, Germany; 4 German Cancer Consortium (DKTK), partner site Dresden and German Cancer Research Center (DKFZ), Heidelberg, Germany; 5 Department of Radiotherapy and Radiation Oncology, Faculty of Medicine and University Hospital Carl Gustav Carus, Technische Universität Dresden, Dresden, Germany; 6 German Cancer Research Center (DKFZ), Heidelberg, Germany; 7 National Center for Tumor Diseases (NCT), partner site Dresden: German Cancer Research Center (DKFZ), Heidelberg; Faculty of Medicine and University Hospital Carl Gustav Carus, Technische Universität Dresden, and Helmholtz-Zentrum Dresden-Rossendorf (HZDR), Dresden, Germany

**Keywords:** Cancer stem cells, Head and neck cancer, Oncogenes, Prognostic markers

## Abstract

Head and neck squamous cell carcinoma (HNSCC) is often being diagnosed at an advanced stage, conferring a poor prognosis. The probability of local tumor control after radiotherapy depends on the eradication of cancer stem cells (CSCs) with activated DNA repair. This study provides evidence that the CSC-related transcription factor Oct4 contributes to HNSCC radioresistance by regulating DNA damage response and the CSC phenotype. Knockdown of Oct4 A isoform reduced self-renewal capacity in HNSCC and led to partial tumor cell radiosensitization caused by transcriptional downregulation of the cell cycle checkpoint kinases CHK1 and WEE1 and homologous recombination (HR) repair genes PSMC3IP and RAD54L. Besides, PARP inhibition with Olaparib selectively radiosensitized Oct4 A knockout, but not wild-type HNSCC cells. This finding links Oct4 A to the HR-mediated DNA repair mechanisms. In turn, knockdown of PSMC3IP and RAD54L reduced the HNSCC self-renewal capacity and clonogenic cell survival after irradiation, suggesting the interplay between DNA repair and the CSC phenotype. Similar to the effect of Oct4 knockdown, overexpression of Oct4 also resulted in significant HNSCC radiosensitization and increased DNA damage, suggesting that Oct4-dependent regulation of DNA repair depends on its fine-tuned expression. In line with this observation, HNSCC patients with high and low nuclear Oct4 expression at the invasive tumor front exhibited better loco-regional tumor control after postoperative radio(chemo)therapy compared to the intermediate expression subgroup. Thus, we found that the Oct4-driven transcriptional program plays a critical role in regulating HNSCC radioresistance, and a combination of radiotherapy with PARP inhibitors may induce synthetic lethality in Oct4-deregulated tumors.

## Introduction

As the sixth most common cancer entity worldwide, head and neck squamous cell carcinoma (HNSCC) accounts for about 900,000 new cases annually [1]. HNSCC displays a high grade of heterogeneity [2]. It can be attributed to the diversity of manifestation sites in the upper aerodigestive tract and the different etiologic backgrounds, including tobacco or alcohol consumption [3] and virus infection [4]. Consequently, the response of patients to multimodal treatment, including surgery and/or radio(chemo)therapy exhibits similar diversity. In most cases, patients with HNSCC are diagnosed with advanced stages of disease, for which a 5-year survival rate of about 50% is being reported [5]. With the concept of personalized medicine evolving, increasing efforts have been made to understand and exploit the molecular mechanisms behind tumor heterogeneity for patient stratification and the development of targeted therapies [6]. For HNSCC arising in the oropharynx, one of the most critical biomarkers established today is the human papillomavirus (HPV) infection status [7]. It identifies a patient subgroup with a good prognosis and a high likelihood of an increased response to radio(chemo)therapy [7, 8]. Still, equally suitable markers for treatment selection and disease monitoring in HPV-negative HNSCC are lacking.

Cancer stem cells (CSCs) have entered the focus of biomarker research due to their self-renewal properties and high radio(chemo)therapy resistance, rendering them a driving force of tumor progression and relapse [9–11]. Several studies reported the applicability of cell surface receptors and intracellular proteins as HNSCC stem cell markers, including the hyaluronic acid receptor CD44 [12], aldehyde dehydrogenase (ALDH) enzymes [13], and transcription factors such as Oct4 [14].

Oct4 is a stemness-associated transcription factor encoded by the *POU5F1* gene. By acting as a transcriptional activator or repressor, Oct4 orchestrates the pluripotency network in human embryonic stem cells (hESCs) [15]. Similarly, Oct4 was shown to promote the self-renewal of CSCs in different tumor entities [16, 17], including HNSCC [14]. However, a comparison between Oct4 target genes in hESCs and germ cell tumors revealed only a partial overlap [18]. It indicates that mechanistic insights regarding the well-characterized functions of Oct4 in hESC might not always contribute to its role in CSCs. Moreover, the plasticity and heterogeneity of CSC subpopulations further complicate the detailed understanding of Oct4-related signaling [19–21]. In the context of tumor progression and radio(chemo)therapy resistance, Oct4 was shown to be involved in the regulation of cellular growth and proliferation, cell cycle control, epithelial-mesenchymal transition (EMT), and DNA repair [17, 22, 23]. Importantly, most studies are lacking the discrimination between the different Oct4 isoforms A, B, and B1, although recent findings suggest their involvement in the various cellular processes. Oct4 isoform A acts as a pluripotency transcription factor, whereas Oct4 B cannot maintain the stem cell phenotype [24]. Instead, there is evidence that Oct4 B probably functions in response to genotoxic agents and other external stressors, which can induce expression of Oct4 B protein variants by alternative translation initiation [25, 26]. Despite many proposed mechanisms in different tumor entities, the role of Oct4 isoforms in the regulation of HNSCC radioresistance remains to be elucidated.

In this report, we provide evidence for the suitability of Oct4 as a biomarker for HNSCC patients treated with postoperative radio(chemo)therapy. With particular emphasis on the different isoforms, we investigate the contribution of Oct4 and the Oct4-related gene signature to HNSCC radioresistance and CSC properties via the regulation of homologous recombination DNA repair.

## Results

Additional results not described here are included in the supplementary information.

### Oct4 depletion is associated with altered CSC properties and DNA damage response

**Fig. 1:**
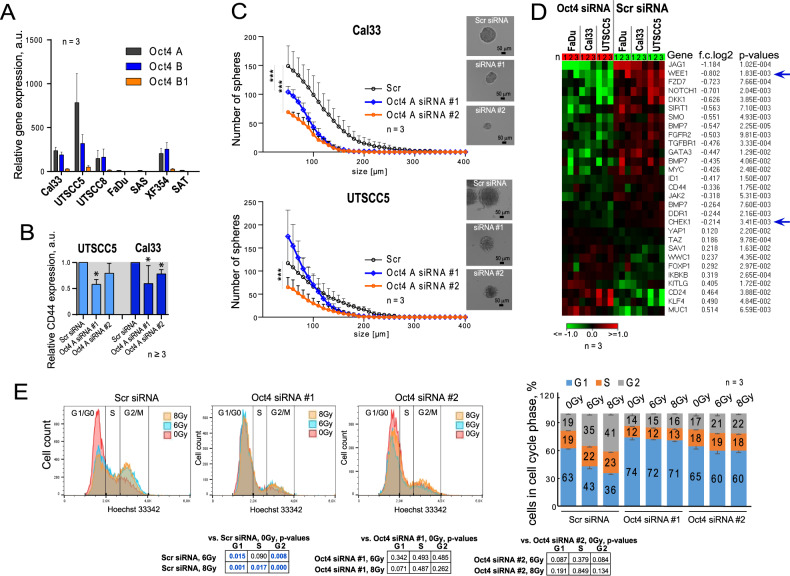
Expression levels of Oct4 regulates CSC phenotype and irradiation-induced G2 arrest. **A** Quantitative real-time PCR (RT-qPCR) analysis of Oct4 A, Oct4 B, and Oct4 B1 expression in seven HNSCC cell lines. **B** Expression of *CD44* in HNSCC cells after Oct4 knockdown; error bars indicate SD; *p < 0,05. **C** Analysis of sphere-forming properties of Cal33 cells after small interfering (si) RNA-mediated knock-down of Oct4 A expression in Cal33 and UTSCC5 cells. Cells transfected with scrambled (Scr) siRNA were used as control; error bars = SD; ***p < 0,001. **D** The whole-genome gene expression analysis of Cal33, FaDu, and UTSCC5 cells transfected with POU5F1 siRNA or scrambled siRNA revealed that Oct4 downregulation is associated with a decrease in CSC-related gene expression. The cell cycle and DNA damage response regulators, WEE1 and CHK1 kinases, are indicated by arrows. **E** Oct4 knockdown in UTSCC5 cells led to the abrogation of the irradiation-induced G2-arrest.

The local tumor control after fractionated irradiation depends on the pre-treatment number of CSCs and their intrinsic radioresistance [27, 28]. As Oct4 expression is associated with pluripotency in CSCs [29–31], we hypothesized that Oct4 might exert its role in regulating HNSCC radioresistance via maintenance of the CSC phenotype. The CSC phenotype in HNSCC is characterized by the expression of several biomarkers, including the hyaluronic acid receptor CD44 as well as the ALDH isoforms ALDH1A1 and ALDH1A3 [32, 33]. To analyze CSC biomarker expression upon Oct4 downregulation, we employed the HPV-negative HNSCC cell lines UTSCC5 and Cal33, which exhibit high Oct4 mRNA levels (Fig. 1A). Discussion of Oct4 isoforms and more detailed analysis of Oct4 mRNA and protein expression in HNSCC cell lines are provided in supplementary results (Supplementary Fig. 1A, B). Indeed, treatment with siRNA against Oct4 A decreased CD44 gene expression in Cal33 and UTSCC5 cells (Fig. 1B and Supplementary Fig. 1C). Besides, the expression of ALDH isoforms ALDH1A1 and ALDH1A3 was markedly deregulated in a cell line- and siRNA-specific manner (Supplementary Fig. 1D). These results suggest that Oct4 downregulation influences the CSC phenotype in HNSCC cell lines. Moreover, Oct4 expression is associated with tumor sphere formation capability of cancer cell lines [34]. To test if Oct4 regulates functional CSC characteristics in HNSCC cell lines, we assessed the sphere-forming capacity of UTSCC5 and Cal33 cells upon siRNA-mediated Oct4 knockdown. In the Cal33 cell line, the reduction of Oct4 expression led to a significant decrease in average sphere size (Fig. 1C and Supplementary Fig. 1C), indicating the involvement of Oct4 in the regulation of self-renewal. Furthermore, in HNSCC cell lines with total Oct4 knockdown, a whole-genome gene expression analysis revealed deregulation in the expression of CSC-related genes (Fig. 1D). Among the CSC genes downregulated by Oct4 knockdown, we identified mediators of various cell signaling pathways, including Wnt/ß-Catenin, NOTCH, ERK, and TGF-ß signaling, as well as cell cycle regulators, like WEE1 G2 Checkpoint Kinase (*WEE1*) and CHK1, checkpoint kinase 1 (*CHEK1*). WEE1 and CHK1 play a pivotal role in DNA damage response (DDR) activated in the CSC populations from different tumor entities [35–37].

A qRT-PCR analysis of *CHEK1* and *WEE1* mRNA expression upon Oct4 isoform A knockdown confirmed the downregulation of both genes in UTSCC5 and Cal33 cells (Supplementary Fig. 2A). In line with the reduced mRNA expression, CHK1 protein levels are decreased in UTSCC5, Cal33, and FaDu cells in response to Oct4 A knockdown, as seen by western blot (Supplementary Fig. 2B). As CHK1 and WEE1 are major regulators of the DNA-damage-induced cell cycle arrest in the G2 phase [38, 39], we investigated the effect of Oct4 knockdown on cell cycle distribution in UTSCC5 cells after irradiation. Whereas UTSCC5 cells treated with scrambled siRNA exhibited a marked increase in G2 phase cells after 6 Gy and a less pronounced upregulation of the S phase cells after 8 Gy, irradiation did not significantly change the cell cycle distribution in Oct4 knockdown cells. There was also no significant difference in the cell cycle distribution for the sham-irradiated cells (Fig. 1E). Consistent with this data, the analysis of the *CHEK1* and *WEE1* gene promoters revealed putative Oct4 binding elements (Supplementary Fig. 2C). These results are consistent with previous observations that HNSCC CSCs defined by side population^+^/CD44^+^/ALDH^high^ phenotype have prolonged G2/M phase arrest in response to carbon or photon irradiation [40]. Our findings indicate that Oct4 is involved in the irradiation-induced DNA damage response and contributes to the regulation of the radioresistant CSC populations.

### Oct4 isoform knockdown partially radiosensitizes HNSCC cell lines

**Fig. 2:**
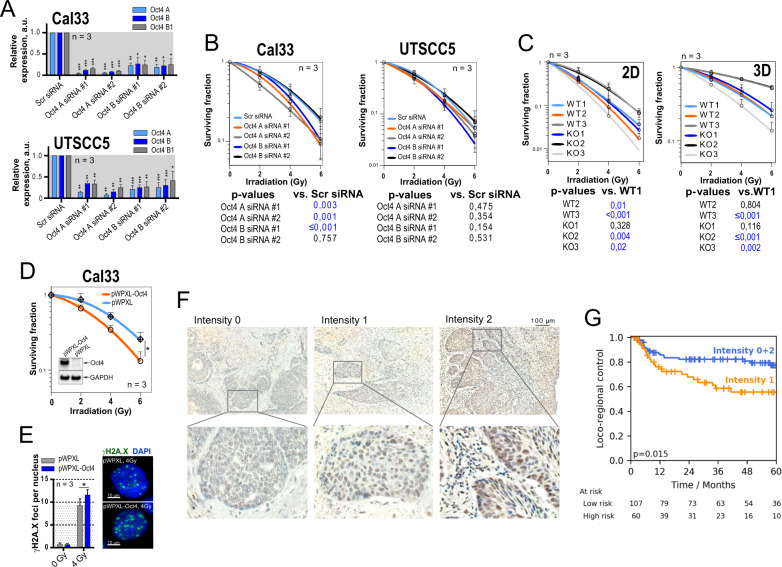
Analysis of the impact of Oct4 expression on HNSCC radioresistance. **A** Relative mRNA expression of Oct4 transcript variants in response to the isoform-specific Oct4 gene knockdown analyzed by RT-qPCR. Cells transfected with scrambled (Scr) siRNA were used as control; error bars indicate SD; *p < 0,01, **p < 0,01, ***p < 0,001. **B** Relative cell radiosensitivity was analyzed by 2D radiobiological colony-forming assay after siRNA-mediated knockdown of Oct4 A or Oct4 B in Cal33 or UTSCC5 cells. Cells transfected with scrambled (Scr) siRNA were used as control; error bars indicate SD. **C** Analysis of relative radioresistance of wild type (WT) and Oct4A knockout (KO) clones of UT SCC5 cells by 2D or 3D colony-forming assay; error bars indicate SD. **D** Radiobiological colony formation analysis of Cal33 cells stably transfected with pWPXL-tdTomato or pWPXL-Oct4-HA-tdTomato plasmids. Expression of Oct4 protein was confirmed by Western blotting; error bars indicate SD; *p < 0,05. **E** The overexpression of Oct4 protein resulted in the accumulation of DNA double-stranded breaks (DSBs) after irradiation. DNA DSBs were analyzed by γ-H2A.X foci analysis 24 h after 4 Gy of X-ray irradiation. Sham-irradiated cells were used as control; error bars indicate SD; *p < 0,05. **F** Representative examples of Oct4 nuclear staining at the invasive tumor front in HNSCC tissues scored as low, intermediate, and high Oct4 expression. The scale bar is 100 µm. **G** Kaplan–Meier analysis of patients treated with postoperative radio(chemo)therapy (PORT-C). The impact of Oct4 expression on loco-regional control was evaluated using the univariate Cox-regression model. Statistical analysis was performed by SPSS software. High and low nuclear Oct4 expression at the invasive front is associated with better loco-regional control; *n* = 167.

To test whether Oct4 is functionally involved in the regulation of HNSCC radioresistance, we employed a siRNA-mediated knockdown of the Oct4 isoforms in the UTSCC5 and Cal33 cell lines. We found that the siRNAs designed to target specifically Oct4 isoform A or B also decreased the expression of all other isoforms, creating an effect resembling total Oct4 knockdown (Fig. 2A). This observation could be explained by a regulatory impact of specific Oct4 isoforms on the expression of other transcript variants, as it was already shown for Oct4 A and B [41]. Furthermore, siRNAs employed in this experiment can potentially target non-spliced premature mRNA, affecting all splice variants. In a 2D colony formation assay, a significant reduction of clonogenic survival after irradiation was observed in Cal33 cells upon Oct4 isoform knockdown (Fig. 2B and Supplementary Fig. 3A, B). In contrast, the UTSCC5 cell line revealed to be less susceptible to Oct4 knockdown-induced radiosensitization. These findings suggested a cell-line-dependent effect of Oct4 knockdown on the HNSCC cell radiosensitivity.

### CRISPR/Cas9-mediated knockout of Oct4 isoform A heterogeneously affects relative cell radioresistance

The generation of the CRISPR/Cas9-mediated knockout of Oct4 isoform A (OCT4 A KO) is described in supplementary results (Supplementary Fig. 4A, B). Assessment of the clonogenic survival after irradiation revealed a heterogeneous effect of Oct4 A KO on relative cell radioresistance (Fig. 2C and Supplementary Fig. 4C). Oct4 A KO clones were among the most radioresistant clones (KO #2), as well as among the most radiosensitive (KO #3). A repetition of the assay under more physiological, Matrigel-embedded conditions (3D colony formation assay) led to very similar results (Fig. 2C and Supplementary Fig. 4C). As HNSCC cell lines possess a high genetic and epigenetic heterogeneity [42, 43], the diverse radioresistance levels of the KO and WT clones may also be attributed to the clone effect. Consequently, the established model systems did not confirm a crucial impact of Oct4 depletion on HNSCC radioresistance in vitro. Instead, our findings suggest that Oct4 downregulation contributes to the regulation of HNSCC radioresistance in a context-dependent manner and potentially can exert its effects in interplay with other signaling molecules, which are differently regulated in the analyzed experimental models.

### Oct4 overexpression results in cell radiosensitization

To further analyze the impact of Oct4 deregulation on HNSCC radioresistance, we generated Cal33-tdTomato and Cal33-Oct4-HA-tdTomato cell lines by stable transfection of Cal33 cells with pWPXL-tdTomato and pWPXL-Oct4-HA-tdTomato plasmids. Analysis of these cell lines for relative clonogenic survival after irradiation showed that Oct4 overexpression results in significant cell radiosensitization (Fig. 2D and Supplementary Fig. 4D). Cal33-Oct4 overexpressing cells exhibit a significantly increased number of residual y-H2A.X foci per nucleus 24 h after irradiation (Fig. 2E). Furthermore, stable Oct4 overexpression is associated with significant downregulation of *BRCA1* and upregulation of *CD44* gene expression (Supplementary Fig. 4E). A high level of Oct4 expression also resulted in the inhibition of *RAD54L*, *SOX2,* and *NANOG* expression (not significant). Altogether, these findings suggest that overexpression of Oct4, like its genetic silencing, causes tumor cell radiosensitization. This finding is in line with the previous observations that distinct cellular responses induced by Oct4 dependent on its intracellular amount. Both up- and downregulation of Oct4 lead to the inhibition of the stem cell transcriptional program [44, 45].

### Increased loco-regional control after postoperative radio(chemo)therapy is associated with Oct4 expression

The expression of CSC-associated genes was recently linked to malignant progression and therapy resistance of patients with HNSCC [32], and in particular to respond to radiotherapy [27, 46]. Here, we investigated a possible association of Oct4 protein expression with loco-regional control in a retrospective, monocentric cohort including 167 patients with locally advanced HNSCC who received cisplatin-based postoperative radio(chemo)therapy (PORT-C). According to the intensity of immunohistochemical staining of nuclear Oct4 protein at the invasive tumor front, the patients were assigned to high, intermediate, or low Oct4 expressing subgroups. Patients with high and low nuclear Oct4 expression at the invasive tumor front exhibited better loco-regional tumor control compared to the intermediate expression subgroup (Fig. 2F, G, and Table 1). In addition, when the high and low Oct4 expression subgroups were analyzed separately, we found that patients with low Oct4 expression exhibited significantly better loco-regional control compared to the intermediate expression subgroup. The same effect was visible for high Oct4 expression compared to the intermediate expression subgroup, but due to the lower patient number the difference was not statistically significant (Supplementary Fig. 5A). This observation is in line with our in vitro finding that both loss and overexpression of Oct4 may lead to tumor cell radiosensitization. All in all, our findings suggest that Oct4 can be regarded as a potential biomarker for HNSCC patients treated with postoperative radio(chemo)therapy.

**Table 1:** Patient characteristics.

Parameter	Number of patients	%
	In total: 167 patients, treated in 1999–2009	
Gender		
Female/male	22/145	13.2/86.8
Clinical T stage		
1/2/3/4	37/73/33/24	22.2/43.7/19.8/14.4
Clinical N stage		
0/1/2/3	16/31/118/2	9.6/18.6/70.7/1.2
UICC stage (2010)		
I/II/III/IV	2/2/33/130	1.2/1.2/19.8/77.8
R status		
0/1/unknown	121/36/10	72.5/21.6/6.0
ECE status		
0/1	98/69	58.7/41.3
Localization		
Oropharynx/oral cavity/hypopharynx/larynx	50/90/20/7	29.9/53.9/12.0/4.2
Grading		
1/2/3	2/78/87	1.2/46.7/52.1
Chemotherapy		
Yes/no	76/91	45.5/54.5
Smoking during therapy		
Yes/no/unknown	121/17/29	72.5/10.2/17.4
Alcohol during therapy		
Yes/no/unknown	117/20/30	70.1/12.0/18.0
p16 status		
Negative/positive/unknown	137/29/1	82.0/17.4/0.6
HPV16 DNA status		
Negative/positive/unknown	138/28/1	82.6/16.8/0.6
Parameter	Median	Range
Follow-up (months)	43.2	1.8–153.0
Age (years)	52.7	24.0–73.0
Dose (Gy)	64.0	60.0–66.0

### PARP inhibition selectively radiosensitizes Oct4 A depleted UTSCC cells

Next, we investigated the molecular mechanisms mediating the role of Oct4 in the regulation of tumor cell radiosensitivity. Our previous studies showed that the UTSCC5 cell line, which was not significantly radiosensitized by Oct4 depletion, is characterized by a substantially higher level of homologous recombination than Cal33 cells, which showed significant radiosensitization upon Oct4 silencing (Fig. 2B) [47]. It was also previously demonstrated that cells with defective HR mechanisms are highly sensitive to the inhibition of Poly (ADP-ribose) polymerase (PARP) [48]. Gene expression analyses revealed that Oct4 depletion resulted in the downregulation of genes involved in homologous DNA repair, such as checkpoint kinases *CHEK1* and *WEE1* (Fig. 1D). Checkpoint kinases are activated in response to irradiation-induced DNA damage to prevent the entry of the damaged cells into mitosis, allowing DNA repair to occur. Moreover, both *CHEK1* and *WEE1* can directly influence homologous recombination repair by regulating phosphorylation of RAD51 and BRCA2, respectively [49, 50].

**Fig. 3:**
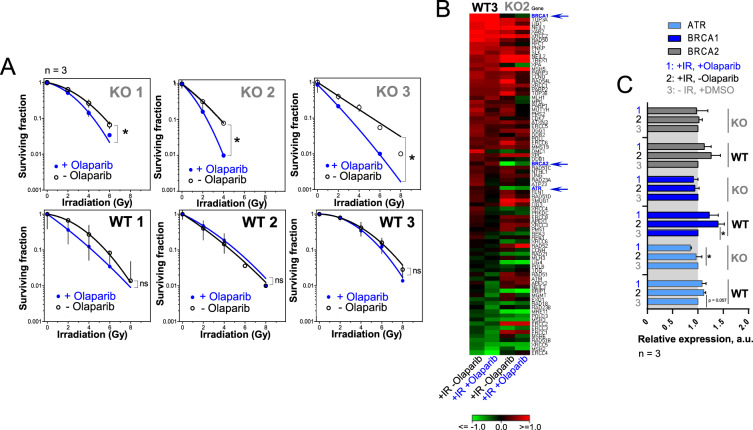
PARP inhibition selectively radiosensitizes Oct4 A depleted HNSCC cells. **A** Radiobiological colony formation assay using Oct4 A KO and WT clones pretreated with Olaparib at a concentration of 1 µM for 2 h before irradiation; error bars indicate SD; *p < 0,05. **B** Expression of 83 DNA repair genes analyzed by RT² Profiler PCR array in UTSCC5 Oct4 A KO and WT clones. Cells were pretreated with Olaparib at a concentration of 1 µM for 2 h before irradiation with 4 Gy of X-ray and collected 24 h later. Data are from three pooled experiments. Gene expression data for Oct4 KO and WT clones are normalized to the corresponding DMSO treated control. **C** Validation of the PCR array results for *ATR*, *BRCA1,* and *BRCA2* genes.

To further investigate a possible contribution of homologous recombination repair to the radiosensitizing effects of Oct4 depletion, we assessed colony formation capability in Oct4 A KO cells with and without treatment with PARP inhibitor Olaparib (Fig. 3A and Supplementary Fig. 5B). While Olaparib reduced plating efficacy at 0 Gy in both Oct4 A KO and wild type clones, the cytotoxic effect was more pronounced in Oct4 A KO cells. Moreover, when Olaparib treatment was followed by irradiation, the PARP inhibition led to radiosensitization only in the Oct4 A KO clones, whereas wild-type clones were not significantly affected. The different susceptibility towards PARP inhibition indicates a HRR deficiency in Oct4 A KO clones. To assess a putative link between Oct4 and HRR mechanisms, we analyzed the expression of 83 DNA repair genes in a representative UTSCC5 Oct4 A KO clone and the corresponding WT clone after irradiation in combination with Olaparib (Fig. 3B). Irradiation alone led to an increased expression of most DNA repair genes in the WT clone, whereas in the Oct4 A KO clone this response was partially disrupted. The addition of Olaparib reduced irradiation response in both Oct4 A KO and WT cells. Among the genes differently regulated between Oct4 A KO and WT cells upon irradiation, we identified ATR, an apex mediator of the DNA damage response, and the HRR genes BRCA1 and BRCA2. qPCR analysis confirmed that BRCA1 is significantly and ATR is close to significantly upregulated upon irradiation only in the WT, but not the Oct4 A KO cells, supporting the hypothesis of a deficiency in DNA repair mechanisms caused by Oct4 A depletion (Fig. 3C). A potential adaptation of UTSCC5 KO cells to the long-term Oct4 depletion and its possible molecular mechanisms are discussed in supplementary results (Supplementary Fig. 5C, D). Altogether, these findings suggest a synthetic lethality effect from the combination of Oct4 A KO and PARP inhibition and involvement of Oct4 A in the homologous recombination repair mechanisms.

### Analysis of the Oct4-related signature confirms involvement in DNA repair and identifies new target genes

**Fig. 4:**
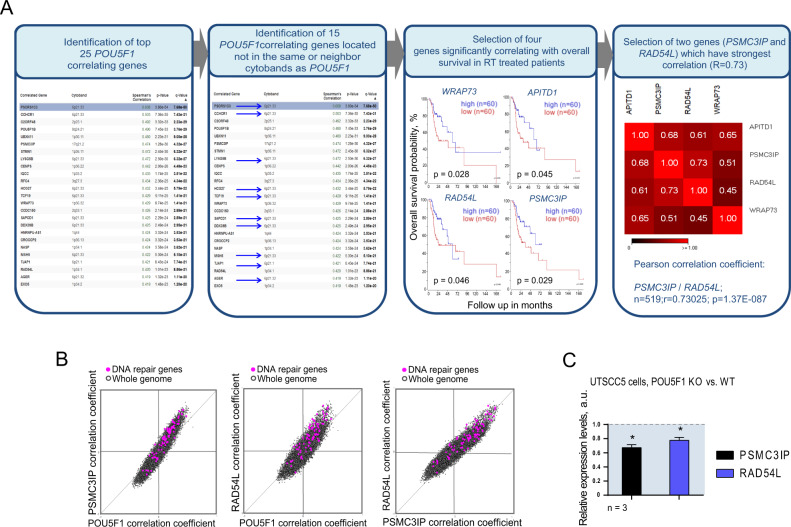
Identification of RAD54L and PSMC3IP as Oct4-correlating genes. **A** Identification of the Oct4-correlating genes in a TCGA HNSCC patient cohort, *n* = 519. *R* value was determined using Pearson correlation test. The blue arrows indicate genes located in the same or neighbor cytobands, which were not selected for further analysis. **B** Correlation of mRNA expression for 83 DNA repair genes with PSMC3IP, RAD54L, and POU5F1 in the TCGA HNSCC patient cohort. **C** Expression of *PSMC3IP* and *RAD54L* genes in UTSCC5 Oct4 A KO and WT clones; error bars indicate SD; *p < 0,05.

**Fig. 5:**
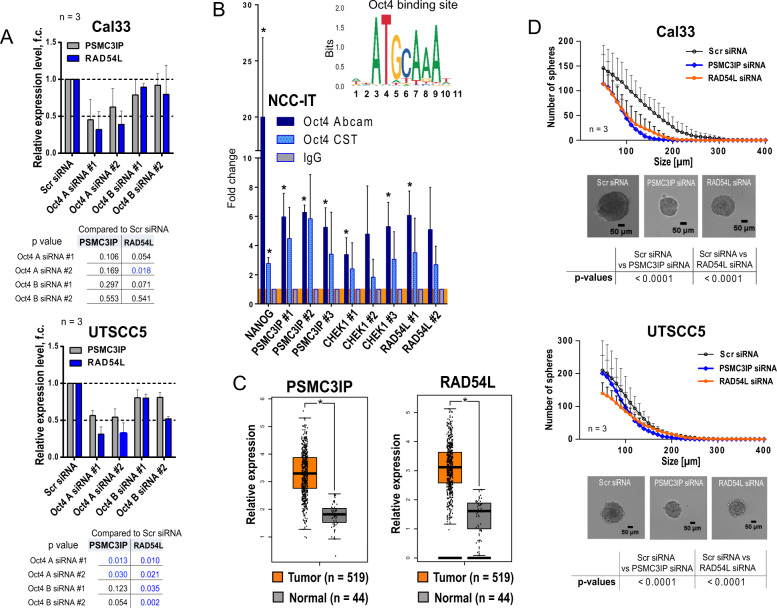
PSMC3IP and RAD54L genes are associated with CSC properties in HNSCC cells and are highly expressed in tumor tissues. **A** Downregulation of *PSMC3IP* and *RAD54L* mRNA levels after siRNA-mediated knockdown of Oct4 A or Oct4 B gene expression in Cal33 and UTSCC5 cells. Cells transfected with scrambled (Scr) siRNA were used as control; error bars indicate SD. **B** The results of chromatin immunoprecipitation (ChIP)–qPCR analysis confirmed direct binding of Oct4 protein to the multiple promoter regions of the target genes *PSMC3IP*, *RAD54L* and *CHEK1*. For this analysis, we used two Oct4 antibodies (Cell Signaling Technology, #2750, and Abcam #ab19857). Rabbit IgG was used as negative control; error bars indicate SD; *p < 0,05. **C** Analysis of the expression levels of *PSMC3IP* and *RAD54L* genes in HNSCC and normal tissues using GEPIA (Gene Expression Profiling Interactive Analysis); *p < 0.05. **D** Downregulation of sphere-forming properties of Cal33 and UTSCC5 cells after siRNA-mediated knockdown of *PSMC3IP* and *RAD54L* gene expression. Cells transfected with scrambled (Scr) siRNA were used as control; error bars indicate SD.

To gain further insight into the Oct4-related gene signature, we performed mRNA co-expression analysis of Oct4 with the whole transcriptome (20 000 genes) using the TCGA HNSCC provisional dataset. Among the 25 genes most correlating with Oct4, we further selected 15 genes that were not located in the same or neighboring cytoband to separate a possible chromatin effect on the gene expression from the functional interactions. In the next step, we identified four genes that showed a significant association with overall survival of HNSCC patients treated with radiotherapy, namely PSMC3IP (HOP2), RAD54L, WRAP73, and APITD1 (Fig. 4A). From this list, we selected PSMC3IP and RAD54L for further analysis, as they exhibit a high correlation not only to Oct4 but also to each other, suggesting an involvement in the same functional cluster. This assumption is supported by the fact that both PSMC3IP and RAD54L are important contributors to homologous recombination repair [51, 52], and have a higher association with overall survival in patients treated with radiotherapy as compared to the total HNSCC TCGA cohort of patients who received heterogenous treatment (Supplementary Fig. 6A and B). In further support of the link between the Oct4-related gene signature and radioresistance, we found a strong correlation of a set of 83 DNA repair genes and Oct4, PSMC3IP and RAD54L in the HNSCC TCGA patient dataset (Fig. 4B). Moreover, co-expression analysis of PSMC3IP and RAD54L with CHEK-1, which is one of the Oct4-regulated genes (Fig. 1D), identified the clusters of genes highly correlating with all three targets (Supplementary Fig. 7A). Pathway analysis revealed a high involvement of these genes in DNA repair and cell cycle regulation mechanisms (Supplementary Fig. 7B and C), highlighting the correlation of Oct4, PSMC3IP, and RAD54L with the DNA damage response. Interestingly, gene expression of PSMC3IP and RAD54L in a UTSCC5 Oct4 A KO clone presumably harboring a homologous recombination repair (HRR) deficiency was significantly lower than in the corresponding WT clone (Fig. 4C). Moreover, both genes are downregulated in response to the siRNA-mediated knockdown of Oct4 (Fig. 5A). Taken together with the occurrence of putative Oct4 binding elements in the promoter of both genes (Supplementary Fig. 8A), this finding suggests that PSMC3IP and RAD54L might be direct targets of Oct4 transcription factor activity.

### PSMC3IP, RAD54L, and CHEK-1 are direct Oct4 target genes with sequential expression profiles

PSMC3IP, RAD54L and CHEK-1 harbor putative Oct4-binding elements in their promoter (Supplementary Fig. 2A and Supplementary Fig. 8A), raising the question whether Oct4 directly regulates transcription of the DNA damage response genes. To this end, we performed Chromatin Immunoprecipitation (ChIP) analysis with two different ChIP-grade antibodies directed against total Oct4 protein. Coverage of all predicted binding sites was achieved by employing multiple primer pairs for each gene promotor. A previously validated Oct4 binding site in the NANOG promoter was used as positive control [53]. Due to the rather low Oct4 protein expression in cancer cell lines [54], including HNSCC cells, this experiment was conducted in the pluripotent embryonal carcinoma cell line NCCIT, which highly expressed Oct4 isoform A and the putative Oct4 target genes (Supplementary Fig. 8B). Our analysis revealed significantly increased precipitation of different promotor regions of NANOG, PSMC3IP, RAD54L and CHEK-1 with Oct4 antibody compared to control IgG (Fig. 5B). These findings suggest that the identified genes are indeed direct targets of Oct4 transcription factor activity.

To further understand Oct4-dependent transcriptional regulation in HNSCC, we performed a time-course analysis of PSMC3IP, RAD54L and CHEK-1 expression upon siRNA-mediated Oct4 knockdown in the Cal33 cell line (Supplementary Fig. 8C). Interestingly, while PSMC3IP mRNA levels decreased as early as 12 h after transfection, RAD54L and CHEK-1 expression was significantly downregulated after 24 h and 48 h, respectively. The observed dynamics indicate a sequential regulation of Oct4 target genes in HNSCC cells potentially driven by additional transcriptional regulators and Oct4-binding partners [15]. Correlation of POU5F1, RAD54L and PSMC3IP expression with genomic instability and HPV16 status is discussed in supplementary results (Supplementary Fig. 9 and Supplementary Table 1).

### PSMC3IP and RAD54L functionally contribute to HNSCC self-renewal and radioresistance

**Fig. 6:**
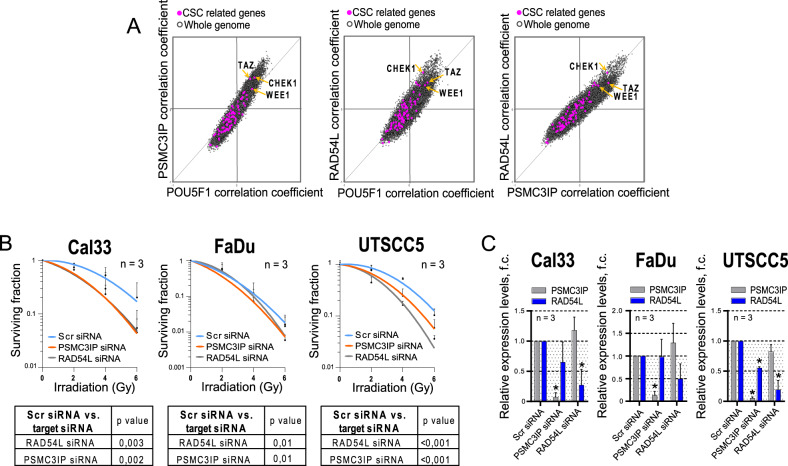
PSMC3IP and RAD54L genes regulate HNSCC radioresistance. **A** Correlation of mRNA expression for 77 CSC-related genes with *PSMC3IP*, *RAD54L*, and *POU5F1* in the TCGA HNSCC patient cohort (*n* = 519) revealed the highest correlation with *TAZ*, *CHEK1,* and *WEE1* (indicated by arrows). **B** The siRNA - mediated knockdown of *PSMC3IP* or *RAD54L* expression increased radiosensitivity in HNSCC cell lines Cal33, UT SCC5, and FaDu. Cells transfected with scrambled (Scr) siRNA were used as control; error bars = SD. **C** Quantitative real-time PCR (RT-qPCR) analysis of *PSMC3IP* and *RAD54L* expression in Cal33, FaDu, and UTSCC5 cell lines after siRNA-mediated knockdown. Error bars indicate SD; *p < 0,05.

**Fig. 7:**
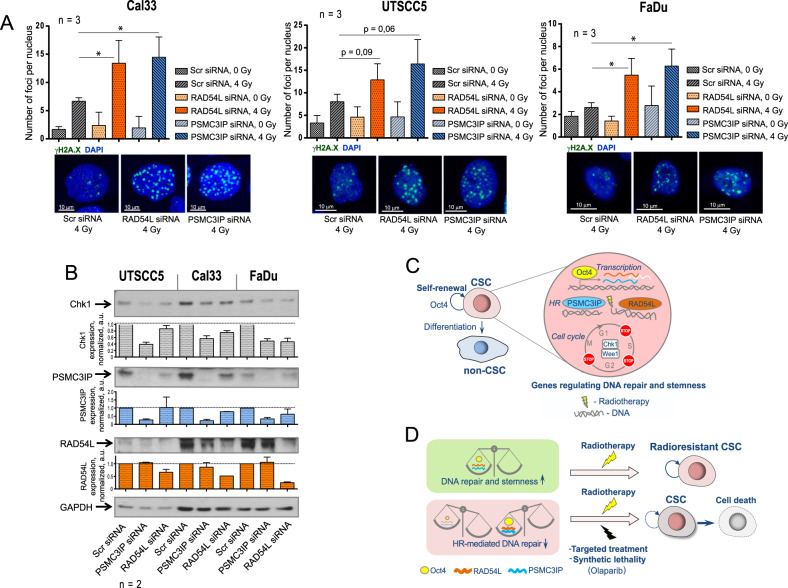
PSMC3IP and RAD54L genes regulate DNA repair. **A** The siRNA-mediated inhibition of PSMC3IP or RAD54L expression resulted in the accumulation of DNA double-stranded breaks (DSBs) after irradiation. DNA DSBs were analyzed in Cal33, UTSCC5, and FaDu cells by γ-H2A.X foci analysis 24 h after 4 Gy of X-ray irradiation. Sham-irradiated cells were used as control; error bars indicate SD; *p < 0,05. The scale bar is 10 µm. **B** Western blot analysis of CHK1, PSMC3IP, and RAD54L expression after siRNA-mediated inhibition of PSMC3IP or RAD54L expression in Cal33, UTSCC5, and FaDu cells. **C** Oct4 contributes to the HNSCC radioresistance by modulating expression of genes regulating DNA repair and CSC properties. **D** Oct4-dependent regulation of tumor radioresistance depends on its precise intracellular level, and both high expression and loss of Oct4 are associated with abnormal homologous recombination (HR)-mediated DNA repair. Combination of radiotherapy with DNA repair inhibitors such as PARP-inhibitors or other targeted treatments may cause synthetic lethality in Oct4-deregulated tumors and CSC death. CSC cancer stem cells, HR homologous recombination.

The potential role of PSMC3IP and RAD54L in tumor development is supported by significantly higher expression levels of these genes in HNSCC than in normal tissues (Fig. 5C). Genetic depletion of both genes affected the self-renewal capacity of HNSCC cell lines. In the sphere formation assay, the siRNA-mediated knockdown of PSMC3IP and RAD54L resulted in a significant decrease in sphere average size and sphere number for Cal33 and UTSCC5 cells (Fig. 5D and Supplementary Fig. 10). Analysis of the 77 CSC-related genes revealed a strong correlation of *POU5F1*, *PSMC3IP*, and *RAD54L* with *CHEK-1*, *WEE1*, and *TAZ*, the critical regulators of the homologous recombination, cell cycle, and CSC maintenance in the HNSCC TCGA patient dataset (Fig. 6A). Several observations validated the role of *PSMC3IP* and *RAD54L* in HNSCC radioresistance regulation and DNA repair. First, siRNA-mediated knockdown of *PSMC3IP* and *RAD54L* expression in Cal33, FaDu, and UTSCC5 cells significantly decreased clonogenic cell survival after irradiation in all analyzed cell lines (Figs. 6B and C, Supplementary Fig. 11). Prompted by the important contribution of both proteins to DNA repair, we analyzed the effect of *PSMC3IP* and *RAD54L* knockdown on y-H2A.X foci clearance as a marker of DNA double-strand break (DSB) repair capacity. Indeed, in Cal33, FaDu and UTSCC5 cells transfected with siRNA against PSMC3IP or RAD54L, the number of residual y-H2A.X foci per nucleus was significantly increased 24 h after irradiation (Fig. 7A). Furthermore, knockdown of both *PSMC3IP* and *RAD54L* genes is associated with the downregulation of CHK1 protein expression (Fig. 7B).

All in all, these findings suggest that Oct4-regulated genes contribute to the HNSCC radioresistance by modulating the DNA repair and CSC properties (Figs. 7C and D). We found that both high expression and loss of Oct4 are associated with abnormal homologous recombination (HR)-mediated DNA repair and can be used to predict high tumor sensitivity to DNA damaging treatment such as radiotherapy. Combining radiotherapy with DNA repair inhibitors such as PARP-inhibitors or other targeted treatments may induce synthetic lethality in Oct4-deregulated tumors, including CSC eradication.

## Discussion

HNSCC is a highly prevalent cancer with a low survival rate at locally advanced stages of the disease. Adequate treatment selection is complicated by the lack of suitable markers, especially for HPV-negative tumors, which in clinical trials exhibit poor prognosis compared to HPV-positive cases [55, 56]. In our study including 167 patients with locally advanced HNSCC, we found that both very low and very high Oct4 protein expression at the invasive tumor front is associated with better loco-regional tumor control after cisplatin-based postoperative radio(chemo)therapy (PORT-C) compared to intermediate Oct4 protein expression. Previously, Ventelä and colleagues reported reduced survival of patients with Oct4-positive HNSCC compared to Oct4-negative cases after radiotherapy [57]. However, our findings indicate that a more detailed categorization of Oct4 expression into low, intermediate and high expression levels could identify additional subgroups of patients who are at high risk for developing loco-regional recurrences, thus further improving its prognostic value. Importantly, our analysis included patients with both HPV-positive and HPV-negative tumors, suggesting the applicability of Oct4 as a biomarker for HNSCC independent of the HPV status. For further discussion on the correlation of Oct4 expression and HPV status, see supplementary information.

Previous studies demonstrated that loco-regional control upon radiotherapy is associated with the number and intrinsic radioresistance properties of CSCs [27, 29]. Our data and earlier published studies described in more detail in the supplementary discussion indicate that Oct4 contributes to radioresistance of HPV-negative HNSCC through maintenance of the CSC phenotype and that the extent of its influence depends on cell-line specific characteristics, which will be discussed below.

Interestingly, among the CSC genes downregulated by Oct4 knockdown, we identified CHK1 and WEE1, two important mediators of the DNA damage response. CSCs crucially depend on efficient DNA repair to maintain stemness and withstand DNA-damaging treatments. Therefore we hypothesized that Oct4 exerts its role in regulating CSC phenotype and radioresistance by contributing to the DNA damage response [10, 27]. Previous reports showed the association of radioresistance and active HRR in HNSCC cell lines [47, 58]. In the UTSCC5 Oct4 A knockout model, irradiation induced expression of the well-known HRR gene *BRCA1* only in the control wild-type cells. In contrast, upregulation of *BRCA1* and other DNA repair genes upon irradiation was impaired in Oct4 A knockout cells. As HRR-deficient tumors are particularly susceptible to PARP inhibition (PARPi), we investigated a potential synthetic lethality effect of PARPi and HRR deficiency induced by Oct4 A depletion [49]. Indeed, PARPi led to significant radiosensitization of Oct4 A knockout, but not wild type UTSCC5 cells, supporting the hypothesis that Oct4 A depletion impairs HRR function. The finding that Oct4 knockout or knockdown alone is not sufficient to radiosensitize UTSCC5 cells can potentially be attributed to compensation by the intrinsically high HRR capacity of this cell line [12]. Moreover, during long-term culturing Oct4 A knockout clones potentially acquired additional changes leading to the activation of pro-survival mechanisms as described in the supplementary discussion. Although the interplay between Oct4 A and different components of the complex DNA damage response warrants more detailed investigation, the synthetic lethality effect of PARPi in Oct4 A-depleted HNSCC cells opens promising directions for further research on CSC-targeting therapies.

To further elucidate the involvement of Oct4 in the DNA damage response in HNSCC, we analyzed the Oct4-correlating gene signature in the TCGA HNSCC dataset and identified two HRR genes, PSMC3IP and RAD54L that are significantly associated with improved overall survival of HNSCC patients treated with radiotherapy. Previous studies suggest that upregulated expression of HRR genes occurs in DNA-repair deficient tumors as a non-functional compensatory mechanism and, therefore, can potentially serves as prognosticator for the efficiency of DNA-damaging therapies [59, 60]. To validate the suitability of PSMC3IP and RAD54L as potential biomarkers for HNSCC treated with radiotherapy, further analyses are required.

In accordance with their reported function in HRR, siRNA-mediated knockdown of PSMC3IP and RAD54L impaired the repair of irradiation-induced DSB and subsequently radiosensitized HNSCC cell lines [51, 61]. Moreover, PSMC3IP and RAD54L knockdown also affected the self-renewal capacity of Cal33 and UTSCC5 cells, reflecting the generally accepted dependency of the CSC phenotype on efficient DNA repair mechanisms [10, 62]. The association of PSMC3IP, RAD54L, and Oct4 with the CSC DNA damage response is further emphasized by co-expression with the checkpoint kinases CHK1 and WEE1 in the HNSCC TCGA patient dataset. An analysis of the cell cycle distribution in UTSCC5 cells after Oct4 knockdown revealed the abrogation of the irradiation-induced G2-arrest, most likely partially attributed to the downregulation of *CHEK-1* and *WEE1* expression. A similar correlation of Oct4 with CHK1 protein expression and radioresistance has been shown for squamous cell carcinoma of the cervix [63]. These findings suggest that modulation of the ATR/CHK1 signaling axis putatively contributes to the radiosensitizing effect of Oct4 depletion in HPV-negative cell lines. Additional investigation is warranted considering the influence of different TP53 mutation variants with loss-of-function, dominant-negative, and gain-of-function activity on the radioresistance and DNA damage response of HNSCC cell lines.

The role of Oct4 in the regulation of the DNA damage response network is further emphasized by the co-expression of CHK1, PSMC3IP, and RAD54L and various other DNA repair genes and cell cycle regulators in the HNSCC TCGA patient dataset. Interestingly, our results showed a downregulation of PSMC3IP, RAD54L, and CHK1 mRNA levels upon Oct4 knockdown in HNSCC cell lines. Consequently, we addressed the question if these genes are direct targets of the Oct4 transcription factor activity. Indeed, a ChIP analysis revealed significantly increased binding of Oct4 protein to different promoter regions of all three genes in the embryonic carcinoma cell line NCCIT.

Nonetheless, caution should be given to the dramatically different Oct4 protein levels of HNSCC and NCCIT cells, as seen by western blot. Similarly, Zhou and colleagues reported three to four orders of magnitude increase in nuclear Oct4 protein of NCCIT cells compared to HeLa cells [54]. Due to its low abundance, the authors suggested the contribution of Oct4-dependent intermediary transcription factors in somatic cancer cell lines. To investigate a possible hierarchical organization of Oct4-dependent transcriptional regulation in HNSCC, we performed time-course analysis after Oct4 knockdown in Cal33 cells and found that PSMC3IP levels are downregulated first, followed by RAD54L and CHK1. This sequential downregulation reflects the finding that PSMC3IP knockdown decreases RAD54L expression, but not vice versa. Moreover, both PSMC3IP and RAD54L knockdown led to a reduction in CHK1 levels. Interestingly, increasing evidence points towards the previously underestimated role of DNA repair proteins in transcription regulation, including a possible function as transcriptional co-activators [64]. For PSMC3IP, a crucial role in the ATF4-dependent transcription has been described [65]. In addition, previous studies report direct regulation of HRR by CHK1 via RAD51 phosphorylation and RAD54L expression modulation [45, 64]. Taken together, our findings suggest that the role of Oct4 in the orchestration of the DNA damage response in HNSCC includes both direct and indirect effects. To further elucidate its complexity, analysis of a potential transcriptional regulation by DNA repair proteins is warranted.

To our knowledge, this is the first report linking the CSC regulator Oct4 with the expression of key players of the HR-mediated DNA repair mechanisms. We found that both high expression and loss of Oct4 are associated with deficient homologous recombination (HR)-mediated DNA repair and can be further exploited to predict high tumor sensitivity after combination of radiotherapy with DNA repair inhibitors. This report also provides evidence that beyond its prognostic value as a biomarker for patients with HNSCC receiving PORT-C, the involvement of Oct4 in the regulation of the DNA damage response opens new insights into HNSCC radioresistance and stemness and suggests possible strategies for combination treatments with PARP inhibitors.

## Materials and methods

Additional methods not described here are included in the supplementary information. Antibodies, primers, and siRNA oligonucleotides used for the study are described in Supplementary Table 2.

### Patient cohort

In this retrospective, monocentric study, 167 patients with locally advanced HNSCC meeting the following criteria were included: histologically proven squamous cell carcinoma arising from the oral cavity, oropharynx, hypopharynx or larynx, availability of sufficient FFPE biomaterial for biomarker analyses, curatively intended treatment between 1999 and 2009 with postoperative radiotherapy (PORT) or cisplatin-based postoperative radiochemotherapy (PORT-C) according to standard protocols covering the former tumor region and regional lymph nodes (50 Gy) and a boost to the former tumor region and the region of the involved neck nodes (10–16 Gy). Prior to the treatment, all patients had to undergo staging (magnetic resonance imaging or computed tomography of the head and neck region, chest X-ray and abdominal ultrasound) to exclude any patients with distant metastases. The included patients were part of two cohorts that were previously presented to identify and validate biomarkers after PORT-C treatment [66, 67].

For patient without progressive disease a minimum follow-up time of 24 months was required. Additionally, formalin-fixed paraffin-embedded (FFPE) tumor material, radiotherapy treatment plans, computed tomography (CT), magnetic resonance imaging (MRI) or positron emission tomography–CT (PET/CT) images of the location of the recurrent tumors as well as follow-up data of patients had to be available to evaluate the localization of the recurrence. The patient characteristics are presented in Table 1.

Ethical approval for this retrospective analysis of clinical and biological data was obtained from the local Ethics Committee.

## Supplementary information

Supplementary results, supplementary discussion, supplementary methods, supplementary figure legends

Supplementary Table 1

Supplementary Table 2

Supplementary Figure 1

Supplementary Figure 2

Supplementary Figure 3

Supplementary Figure 4

Supplementary Figure 5

Supplementary Figure 6

Supplementary Figure 7

Supplementary Figure 8

Supplementary Figure 9

Supplementary Figure 10

Supplementary Figure 11

## References

[1] Chow LQM (2020). Head and neck cancer. N Engl J Med.

[2] Leemans CR, Snijders PJF, Brakenhoff RH (2018). The molecular landscape of head and neck cancer. Nat Rev Cancer.

[3] Hashibe M, Brennan P, Chuang SC, Boccia S, Castellsague X, Chen C (2009). Interaction between tobacco and alcohol use and the risk of head and neck cancer: pooled analysis in the International Head and Neck Cancer Epidemiology Consortium. Cancer Epidemiol Biomark Prev.

[4] Carpen T, Syrjanen S, Jouhi L, Randen-Brady R, Haglund C, Makitie A (2020). Epstein-Barr virus (EBV) and polyomaviruses are detectable in oropharyngeal cancer and EBV may have prognostic impact. Cancer Immunol Immunother.

[5] Leemans CR, Braakhuis BJ, Brakenhoff RH (2011). The molecular biology of head and neck cancer. Nat Rev Cancer.

[6] Razzouk S (2014). Translational genomics and head and neck cancer: toward precision medicine. Clin Genet.

[7] Ang KK, Harris J, Wheeler R, Weber R, Rosenthal DI, Nguyen-Tan PF (2010). Human papillomavirus and survival of patients with oropharyngeal cancer. N. Engl J Med.

[8] O’Sullivan B, Huang SH, Siu LL, Waldron J, Zhao H, Perez-Ordonez B (2013). Deintensification candidate subgroups in human papillomavirus-related oropharyngeal cancer according to minimal risk of distant metastasis. J Clin Oncol.

[9] Baumann M, Krause M, Hill R (2008). Exploring the role of cancer stem cells in radioresistance. Nat Rev Cancer.

[10] Schulz A, Meyer F, Dubrovska A, Borgmann K. Cancer stem cells and radioresistance: DNA repair and beyond. Cancers (Basel). 2019;11(6):862. 10.3390/cancers11060862 PMC6627210 31234336

[11] Bütof R, Dubrovska A, Baumann M (2013). Clinical perspectives of cancer stem cell research in radiation oncology. Radiother Oncol.

[12] Prince ME, Sivanandan R, Kaczorowski A, Wolf GT, Kaplan MJ, Dalerba P (2007). Identification of a subpopulation of cells with cancer stem cell properties in head and neck squamous cell carcinoma. Proc Natl Acad Sci USA.

[13] Chen YC, Chen YW, Hsu HS, Tseng LM, Huang PI, Lu KH (2009). Aldehyde dehydrogenase 1 is a putative marker for cancer stem cells in head and neck squamous cancer. Biochem Biophys Res Commun.

[14] Koo BS, Lee SH, Kim JM, Huang S, Kim SH, Rho YS (2015). Oct4 is a critical regulator of stemness in head and neck squamous carcinoma cells. Oncogene.

[15] Shi G, Jin Y (2010). Role of Oct4 in maintaining and regaining stem cell pluripotency. Stem Cell Res Ther.

[16] Vaddi PK, Stamnes MA, Cao H, Chen S. Elimination of SOX2/OCT4-associated prostate cancer stem cells blocks tumor development and enhances therapeutic response. Cancers (Basel). 2019;11(9):1331. 10.3390/cancers11091331 PMC6769476 31500347

[17] Ruan Z, Yang X, Cheng W (2019). OCT4 accelerates tumorigenesis through activating JAK/STAT signaling in ovarian cancer side population cells. Cancer Manag Res.

[18] Song B, Kim DK, Shin J, Bae SH, Kim HY, Won B (2018). OCT4 directly regulates stemness and extracellular matrix-related genes in human germ cell tumours. Biochem Biophys Res Commun.

[19] Geissler C, Hambek M, Leinung M, Diensthuber M, Gassner D, Stover T (2012). The challenge of tumor heterogeneity–different phenotypes of cancer stem cells in a head and neck squamous cell carcinoma xenograft mouse model. Vivo.

[20] Kreso A, Dick JE (2014). Evolution of the cancer stem cell model. Cell Stem Cell.

[21] Trosko JE. Cancer prevention and therapy of two types of gap junctional intercellular communication(-)deficient “Cancer Stem Cell”. Cancers (Basel) 2019;11(1):87. 10.3390/cancers11010087 PMC6356618 30646567

[22] Shao M, Bi T, Ding W, Yu C, Jiang C, Yang H (2018). OCT4 Potentiates radio-resistance and migration activity of rectal cancer cells by improving epithelial-mesenchymal transition in a ZEB1 dependent manner. Biomed Res Int.

[23] Kim JY, Kim JC, Lee JY, Park MJ (2018). Oct4 suppresses IRinduced premature senescence in breast cancer cells through STAT3- and NFkappaB-mediated IL24 production. Int J Oncol.

[24] Lee J, Kim HK, Rho JY, Han YM, Kim J (2006). The human OCT-4 isoforms differ in their ability to confer self-renewal. J Biol Chem.

[25] Gao Y, Wei J, Han J, Wang X, Su G, Zhao Y (2012). The novel function of OCT4B isoform-265 in genotoxic stress. Stem Cells.

[26] Wang X, Zhao Y, Xiao Z, Chen B, Wei Z, Wang B (2009). Alternative translation of OCT4 by an internal ribosome entry site and its novel function in stress response. Stem Cells.

[27] Krause M, Dubrovska A, Linge A, Baumann M (2017). Cancer stem cells: radioresistance, prediction of radiotherapy outcome and specific targets for combined treatments. Adv Drug Deliv Rev.

[28] Yaromina A, Krause M, Thames H, Rosner A, Krause M, Hessel F (2007). Pre-treatment number of clonogenic cells and their radiosensitivity are major determinants of local tumour control after fractionated irradiation. Radiother Oncol.

[29] Kim RJ, Nam JS (2011). OCT4 Expression enhances features of cancer stem cells in a mouse model of breast cancer. Lab Anim Res.

[30] Kumar SM, Liu S, Lu H, Zhang H, Zhang PJ, Gimotty PA (2012). Acquired cancer stem cell phenotypes through Oct4-mediated dedifferentiation. Oncogene.

[31] Tai MH, Chang CC, Kiupel M, Webster JD, Olson LK, Trosko JE (2005). Oct4 expression in adult human stem cells: evidence in support of the stem cell theory of carcinogenesis. Carcinogenesis.

[32] Peitzsch C, Nathansen J, Schniewind SI, Schwarz F, Dubrovska A. Cancer stem cells in head and neck squamous cell carcinoma: identification, characterization and clinical implications. Cancers (Basel) 2019;11(5):616. 10.3390/cancers11050616 PMC6562868 31052565

[33] Kurth I, Hein L, Mabert K, Peitzsch C, Koi L, Cojoc M (2015). Cancer stem cell related markers of radioresistance in head and neck squamous cell carcinoma. Oncotarget.

[34] Jung JW, Park SB, Lee SJ, Seo MS, Trosko JE, Kang KS (2011). Metformin represses self-renewal of the human breast carcinoma stem cells via inhibition of estrogen receptor-mediated OCT4 expression. PLoS One.

[35] Manic G, Signore M, Sistigu A, Russo G, Corradi F, Siteni S (2018). CHK1-targeted therapy to deplete DNA replication-stressed, p53-deficient, hyperdiploid colorectal cancer stem cells. Gut.

[36] Bartucci M, Svensson S, Romania P, Dattilo R, Patrizii M, Signore M (2012). Therapeutic targeting of Chk1 in NSCLC stem cells during chemotherapy. Cell Death Differ.

[37] Venkatesha VA, Parsels LA, Parsels JD, Zhao L, Zabludoff SD, Simeone DM (2012). Sensitization of pancreatic cancer stem cells to gemcitabine by Chk1 inhibition. Neoplasia.

[38] Bartek J, Lukas J (2003). Chk1 and Chk2 kinases in checkpoint control and cancer. Cancer Cell.

[39] Smith HL, Southgate H, Tweddle DA, Curtin NJ (2020). DNA damage checkpoint kinases in cancer. Expert Rev Mol Med.

[40] Bertrand G, Maalouf M, Boivin A, Battiston-Montagne P, Beuve M, Levy A (2014). Targeting head and neck cancer stem cells to overcome resistance to photon and carbon ion radiation. Stem Cell Rev Rep.

[41] Meng L, Hu H, Zhi H, Liu Y, Shi F, Zhang L (2018). OCT4B regulates p53 and p16 pathway genes to prevent apoptosis of breast cancer cells. Oncol Lett.

[42] Kagohara LT, Zamuner F, Davis-Marcisak EF, Sharma G, Considine M, Allen J et al. Integrated single-cell and bulk gene expression and ATAC-seq reveals heterogeneity and early changes in pathways associated with resistance to cetuximab in HNSCC-sensitive cell lines. Br J Cancer 2020;123(1):101–13. 10.1038/s41416-020-0851-5 PMC7341752 32362655

[43] Digomann D, Kurth I, Tyutyunnykova A, Chen O, Löck S, Gorodetska I (2019). The CD98 heavy chain is a marker and regulator of head and neck squamous cell carcinoma radiosensitivity. Clin Cancer Res.

[44] Hammachi F, Morrison GM, Sharov AA, Livigni A, Narayan S, Papapetrou EP (2012). Transcriptional activation by Oct4 is sufficient for the maintenance and induction of pluripotency. Cell Rep.

[45] Niwa H, Miyazaki J, Smith AG (2000). Quantitative expression of Oct-3/4 defines differentiation, dedifferentiation or self-renewal of ES cells. Nat Genet.

[46] Linge A, Lohaus F, Löck S, Nowak A, Gudziol V, Valentini C (2016). HPV status, cancer stem cell marker expression, hypoxia gene signatures and tumour volume identify good prognosis subgroups in patients with HNSCC after primary radiochemotherapy: a multicentre retrospective study of the German Cancer Consortium Radiation Oncology Group (DKTK-ROG). Radiother Oncol.

[47] Wurster S, Hennes F, Parplys AC, Seelbach JI, Mansour WY, Zielinski A (2016). PARP1 inhibition radiosensitizes HNSCC cells deficient in homologous recombination by disabling the DNA replication fork elongation response. Oncotarget.

[48] Bryant HE, Schultz N, Thomas HD, Parker KM, Flower D, Lopez E (2005). Specific killing of BRCA2-deficient tumours with inhibitors of poly(ADP-ribose) polymerase. Nature.

[49] Sorensen CS, Hansen LT, Dziegielewski J, Syljuasen RG, Lundin C, Bartek J (2005). The cell-cycle checkpoint kinase Chk1 is required for mammalian homologous recombination repair. Nat Cell Biol.

[50] Krajewska M, Heijink AM, Bisselink YJ, Seinstra RI, Sillje HH, de Vries EG (2013). Forced activation of Cdk1 via wee1 inhibition impairs homologous recombination. Oncogene.

[51] Pezza RJ, Voloshin ON, Volodin AA, Boateng KA, Bellani MA, Mazin AV (2014). The dual role of HOP2 in mammalian meiotic homologous recombination. Nucleic Acids Res.

[52] Heyer WD, Li X, Rolfsmeier M, Zhang XP (2006). Rad54: the Swiss Army knife of homologous recombination?. Nucleic Acids Res.

[53] Rodda DJ, Chew JL, Lim LH, Loh YH, Wang B, Ng HH (2005). Transcriptional regulation of nanog by OCT4 and SOX2. J Biol Chem.

[54] Zhou Y, Chen X, Kang B, She S, Zhang X, Chen C (2018). Endogenous authentic OCT4A proteins directly regulate FOS/AP-1 transcription in somatic cancer cells. Cell Death Dis.

[55] Kimple RJ, Harari PM (2015). The prognostic value of HPV in head and neck cancer patients undergoing postoperative chemoradiotherapy. Ann Transl Med.

[56] Kimple RJ, Smith MA, Blitzer GC, Torres AD, Martin JA, Yang RZ (2013). Enhanced radiation sensitivity in HPV-positive head and neck cancer. Cancer Res.

[57] Ventela S, Sittig E, Mannermaa L, Makela JA, Kulmala J, Loyttyniemi E (2015). CIP2A is an Oct4 target gene involved in head and neck squamous cell cancer oncogenicity and radioresistance. Oncotarget.

[58] Liu C, Liao K, Gross N, Wang Z, Li G, Zuo W (2020). Homologous recombination enhances radioresistance in hypopharyngeal cancer cell line by targeting DNA damage response. Oral Oncol.

[59] Ciccia A, Elledge SJ (2010). The DNA damage response: making it safe to play with knives. Mol Cell.

[60] Pitroda SP, Pashtan IM, Logan HL, Budke B, Darga TE, Weichselbaum RR (2014). DNA repair pathway gene expression score correlates with repair proficiency and tumor sensitivity to chemotherapy. Sci Transl Med.

[61] Akagi K, Li J, Broutian TR, Padilla-Nash H, Xiao W, Jiang B (2014). Genome-wide analysis of HPV integration in human cancers reveals recurrent, focal genomic instability. Genome Res.

[62] Vitale I, Manic G, De Maria R, Kroemer G, Galluzzi L (2017). DNA damage in stem cells. Mol Cell.

[63] Rachmadi L, Siregar NC, Kanoko M, Andrijono A, Bardosono S, Suryandari DA (2019). Role of cancer stem cell, apoptotic factor, dna repair, and telomerase toward radiation therapy response in stage IIIB cervical cancer. Oman Med J.

[64] Fong YW, Cattoglio C, Tjian R (2013). The intertwined roles of transcription and repair proteins. Mol Cell.

[65] Zhang Y, Lin T, Lian N, Tao H, Li C, Li L (2019). Hop2 interacts with ATF4 to promote osteoblast differentiation. J Bone Min Res.

[66] Linge A, Löck S, Gudziol V, Nowak A, Lohaus F, von Neubeck C (2016). Low cancer stem cell marker expression and low hypoxia identify good prognosis subgroups in HPV(-) HNSCC after postoperative radiochemotherapy: a multicenter study of the DKTK-ROG. Clin Cancer Res.

[67] Lohaus F, Linge A, Tinhofer I, Budach V, Gkika E, Stuschke M (2014). HPV16 DNA status is a strong prognosticator of loco-regional control after postoperative radiochemotherapy of locally advanced oropharyngeal carcinoma: results from a multicentre explorative study of the German Cancer Consortium Radiation Oncology Group (DKTK-ROG). Radiother Oncol.

