# Supplementary material for: Oct4 confers stemness and radioresistance to head and neck squamous cell carcinoma by regulating the homologous recombination factors PSMC3IP and RAD54L
Source: Oncogene. 2021 Jun 2;40(24):4214–28. doi: 10.1038/s41388-021-01842-1 (PMC8211562; doi:10.1038/s41388-021-01842-1)
Supplement: Supplementary file 1 — Supplementary results, supplementary discussion, supplementary methods, supplementary figure legends [file 41388_2021_1842_MOESM1_ESM.pdf]

Supplementary Information

Supplementary results

Oct4 isoforms are differentially expressed in HNSCC cell lines

Due to alternative splicing and alternative translation initiation, the *POU5F1* gene can be transcribed into at least three different transcript variants (TV), which in turn are translated into four protein isoforms (Supplementary Figure 1A) [1]. The expression of the different Oct4 isoforms was analyzed in a set of seven established HNSCC cell lines including Cal33, FaDu, SAT, SAS, UTSCC5, UTSCC8 and XF354. mRNA expression of Oct4 isoform A was found in all seven HNSCC cell lines, whereas Oct4 B and Oct4 B1 mRNA could only be detected in four out of seven cell lines (Figure 1A). The *in vitro* mRNA expression levels of Oct4 isoforms vary considerably between the HNSCC cell lines. While the UTSCC5 cell line shows an approximately four-fold higher expression of Oct4 isoform A than any other cell line, only very low amounts of Oct4 A mRNA could be detected in SAT, SAS and FaDu. Interestingly, western blot analysis revealed that protein levels detected by anti-Oct4 antibodies might not reflect well the Oct4 isoform mRNA levels of HNSCC cell lines (Supplementary Figure 1B). A similar discrepancy has already been reported in other tumor entities [1]. In contrast to the qPCR primers designed in this study, commercially available Oct4 antibodies often fail to distinguish different isoforms as well as discriminate Oct4 from other very similar homologs, such as protein product of the POU5F1B gene [2, 3]. Additionally, the observed discrepancy could be explained by the tightly controlled expression regulation of transcription factors both at mRNA and protein levels [4-6]. In this study, we employed the HNSCC cell lines Cal33 and UTSCC5, which show high mRNA levels as well as protein levels detectable by Oct4 antibody (Supplementary Figure 1B).

CRISPR/Cas9-mediated knockout of Oct4 isoform A

Taking into account the transient effect of siRNA-mediated gene silencing, we established a stable model system allowing the analysis of single-isoform depletion. Using the CRISPR/Cas9 system, we generated UTSCC5 cells with a genomic knockout (KO) of the

pluripotency-related isoform Oct4 A (Supplementary Figure 4 A). To achieve that, insertion or deletion (Indel) mutations were introduced into exon 1 of the POU5F1 gene, which is exclusively present in the Oct4 isoform A. These mutations resulted in mRNA with frameshift sequence, whereas the mRNA expression levels of all Oct4 isoforms in the KO clones were not significantly different from the expression levels in two out of three wild type controls (Supplementary Figure 4 B). Exons commonly present in other isoforms, as Oct4 2b, were not targeted. Three complete Oct4 A knockout clones (KO) were selected for further analyses, along with three wild type (WT) clones that underwent the same procedure but did not have any sequence alterations (Supplementary Figure 4 A).

Potential adaptation of UTSCC5 KO cells to the long-term Oct4 depletion

Interestingly, basal mRNA expression of ATR was higher in the Oct4 A KO clone than in the WT clone (Supplementary Figure 5C). Moreover, in the Oct4 A KO cells we observed a trend towards higher protein levels and increased phosphorylation of CHK1 (Supplementary Figure 5D), which is a downstream target of ATR signaling [2], as compared to the WT cells. These results could be explained by a potential adaptation of UTSCC5 KO cells to the long-term Oct4 depletion by deregulation of the DNA repair pathways, suggesting that long-term KO models adapt to missing Oct4 by deregulation of the DNA repair pathways.

Correlation of *POU5F1*, *RAD54L* and *PSMC3IP* expression with genomic instability and HPV16 status

By analyzing the TCGA HNSCC gene expression dataset, we found that *POU5F1, PSMC3IP,* and *RAD54L* are associated with a high fraction genome altered (FGA) index, which is an indicator of genomic instability (Supplementary Figure 9A). It is driven by many factors, including genetic mutations, epigenetic alterations, changes in the tumor microenvironment, and HPV16 infection [3, 4]. Previous studies showed that HPV16 oncoprotein E7 also activates the Oct4 promoter and increases Oct4 gene expression in HNSCC and cervical carcinoma suggesting potential mechanisms for HPV16-mediated CSC

formation and tumor initiation [5, 6]. That study also found that in cervical carcinoma, Oct4 induces proliferation and migration in HPV negative cells whereas inhibits it in HPV positive cells [6]. Our finding indicates significant correlation of HPV positivity and low expression of Oct4 protein at the invasive front (n = 166), although the fraction of HPV positive tumors is low (n = 28) (Supplementary Table 1).

Using the TCGA HNSCC gene expression dataset, we confirmed increased expression levels of *POU5F1, PSMC3IP,* and *RAD54L* in HPV16 DNA-positive tumors (Supplementary Figure 9B). Of note, the significant association of high DNA repair gene expression with better survival can also be found in HPV16 DNA-negative HNSCC (Supplementary Figure 9C). Similar to our observations, previous clinical studies also demonstrated that high expression of DNA repair genes is associated with homology repair deficiency, and could be used to predict a better response to DNA toxic treatments [7, 8]. These studies suggested that high expression of these genes might reflect not a function, but an attempt to compensate for a defective DNA repair pathway. Furthermore, increased expression of specific DNA repair genes might antagonize homology recombination and lead to increased DNA damage after radiotherapy by disrupting the tight regulation required for efficient DNA repair [8, 9]. Most importantly, these studies provided evidence that high expression levels of DNA repair genes might be used to predict high tumor sensitivity to DNA-damaging therapies [8].

Supplementary methods

Cell lines and culture conditions

The *in vitro* experiments were performed on the HPV-negative HNSCC cell lines Cal33 (DMSZ, Germany), FaDu (ATCC, USA) UTSCC5 and UTSCC8 (both Prof. Reidar Grenman, University of Turku, Finnland), XF354 (DKFZ tumor bank, Heidelberg, Germany), SAS and SAT (both JCRB Cell Bank, Osaka, Japan). Cell line identity was confirmed using PCR-single-locus-technology by Eurofins Genomics. All HNSCC cell lines were cultured in Dulbecco´s Modified Eagle Medium (DMEM) with high glucose (4500 mg/L) (Sigma-Aldrich)

supplemented with 10% FBS, 1 mM L-glutamine, 1% HEPES solution and 1% MEM non-essential amino acids (all from Sigma-Aldrich) at 37 °C and 5% CO_2 in a humidified incubator. NCCIT cells used for chromatin immunoprecipitation analysis were purchased from ATCC® and maintained in RPMI medium (PAN-Biotech) with all supplements listed above. Mycoplasma free status was confirmed regularly.

Immunohistochemical staining of Oct-4 and p16

Sections of Formalin-fixed paraffin-embedded (FFPE) primary tumor samples were deparrafinized and antigen retrieval was conducted as described previously [10] with minor modifications. After antigen retrieval (pH 6; Dako) for 35 min at 630 W, sections were incubated with polyclonal rabbit anti-human Oct4-antibody (dilution 1:100; ab18976, Abcam, Cambridge, UK) for 30 min at room temperature. Negative control slides were incubated with corresponding rabbit antibody control (Dako). Blinded samples were semiquantitatively evaluated by two independent observers. Nuclear Oct4 staining was assessed in the whole tumor specimen and its invasive front, and patients were grouped into high, intermediate and low Oct4 expression subgroup based on the staining intensity.

For immunohistochemical staining of p16, the CINtec histology kit (Roche mtm laboratories AG, Basel, CH) was used according to the instructions of the manufacturer and as described previously [11]. Intense staining of at least 70% of the tumour cells was defined as positive staining.

Curves depicting loco-regional control were estimated by the Kaplan-Meier method in the Statistical Package for the Social Sciences (SPSS) v25 software. Statistical significance was calculated with the log-rank test.

DNA extraction and HPV genotyping

DNA extraction and PCR-array based HPV DNA genotyping were performed as described in [11]. Briefly, 5 µm FFPE sections were subjected to DNA extraction using the QIAamp DNA FFPE tissue kit (Qiagen GmbH, Hilden, DE) according to the instructions of the

manufacturer. HPV DNA analyses including determination of the genotype were performed using the LCD-Array HPV 3.5 kit (CHIPRON GmbH, Berlin, DE) as reported in [11].

Radiobiological colony formation assay (CFA)

For analyses of clonogenic cell survival after irradiation, cells were seeded in triplicates into 6-well-plates at a density of 250-2000 cells/well depending on the cell line and treatment. After attachment of the cells, irradiation was performed with doses of 2, 4 and 6 Gy of X-rays. In case of treatment with PARP inhibitor Olaparib, irradiation was performed 2h after addition of 1 µmol Olaparib. Sham irradiated plates were used as controls. After irradiation, cells were kept for 8 to 14 days, until colonies reached a sufficient size corresponding to more than 50 cells. Cells were fixed and stained with crystal violet (Sigma-Aldrich). Counting of colonies was performed manually.

For 3D-CFA, cells were plated into ultra-low attachment 96-well plates (Corning) at a density of 500 cells/well, immersed in a 0.5 mg/ml Matrigel (Corning) DMEM solution. For each condition, 5 technical repeats were plated. 24 h after seeding, an equal amount of medium was added on top of the Matrigel and cells were irradiated as described above. Fixation was performed 8 to 9 days after irradiation by addition of 10% formaldehyde (VWR International) into the medium. Colonies were counted manually. Statistical significance of the difference between cell survival curves was determined in SPSS v25 using stratified linear regression to fit the data into the linear-quadratic formula $S(D)/S(0) = \exp(\alpha D+\beta D2)$, as described by Franken and colleagues [12]. p values < 0.05 were considered statistically significant.

Sphere formation assay

After harvesting, cells were washed once with PBS and then resuspended to single cells in Mammary Epithelial Cell Growth Medium (MEBM) (Lonza) supplemented with B27 (Invitrogen), 4 µg/ml insulin and 1 mM L-glutamine (both Sigma-Aldrich), 20 ng/ml epidermal growth factor (EGF) and 20 ng/ml basic fibroblast growth factor (FGF) (both Peprotech). 2000 cells/well were plated into 24-well ultra-low attachment plates (Corning) in triplicates. Four

days later, additional 500 µl MEBM per well were added and cell clumps were disintegrated by pipetting. Plates were scanned automatically 7 to 13 days after plating using the Celigo S Imaging Cell Cytometer (Brooks). Spheres were discriminated from cell aggregates by a diameter ≥ 50 µm and a roundish shape. Sphere size was determined from images employing the Fiji/ImageJ software [13].

siRNA-mediated knockdown

For knockdown of the Oct4 isoforms A and B as well as PSMC3IP and RAD54L, cells were seeded at a density of $2x10^5$ to $3x10^5$ cells/well into 6-well plates with complete DMEM medium. On the next day, cells were transfected with 12 µl Lipofectamine RNAi MAX (Invitrogen) according to manufacturer's instructions. For Oct4 isoform knockdown, single siRNA and control non-specific scrambled siRNA were used at a concentration of 150 pmol/well. With the help of the siDESIGN Center online tool (Horizon Discovery), siRNA were designed to target either Oct4 isoform A or B without affecting Oct4 pseudogene expression. siRNA oligonucleotides were synthesized by Eurofins. siRNA sequences are provided in Supplementary Table 2. Knockdown of PSMC3IP and RAD54L was performed using 100 pg/well of Smartpool siRNA purchased from Dharmacon. Scrambled smartpool siRNA was used as control. Cells were harvested and plated for further assays 24 h or 48 h after transfection with PSMC3IP and RAD54L siRNA or Oct4 isoform siRNA, respectively. For time course analysis of target gene expression after Oct4 knockdown, samples were collected 8 h, 12 h, 24 h and 48 h after start of transfection. Efficacy of the knockdown was validated by RT-qPCR and Western Blot. Gene expression analysis for Cal33, FaDu and UTSCC5 cells transfected either with Oct4 siRNA (total) or scrambled siRNA was performed using GPL20844 Agilent-072363 SurePrint G3 Human GE v3 8x60K Microarray 039494. Samples were prepared and arrays were processed using standard Agilent protocols as described earlier [14]. Data deposition: all data are MIAME compliant and the raw data have been deposited in the Gene Expression Omnibus (GEO) database, accession no GSE173161.

Oct4 overexpression

Cal33-tdTomato and Cal33-Oct4-HA-tdTomato cell lines were generated via transfection of Cal33 with pWPXL-tdTomato and pWPXL-Oct4-HA-tdTomato plasmids, followed by two rounds of FACS. In the first round, cells expressing tdTomato were separated from non-transfected population and two weeks later the second sorting was performed to separate the stable population from cells that were only transiently transfected and already lost tdTomato expression.

RNA Isolation, cDNA Synthesis and qPCR

Total RNA isolation was performed with the RNeasy Plus Mini Kit (Qiagen GmbH, Hilden, Germany) according to manufacturer`s instructions. Then, cDNA was synthetized using the PrimeScript™ RT reagent Kit (Takara Bio). For each sample, an additional reaction without reverse transcriptase (RT) enzyme was conducted and pooled minus-RT samples were used as controls for qPCR. qPCR was performed using TB Green Premix Ex Taq II (Tli RNase H Plus) (Takara Bio) according to manufacturer`s protocol in the StepOnePlus™ Real-Time PCR System (Applied Biosystems). Primer sequences are provided in Supplementary Table 2. qPCR reactions were conducted in three technical replicates and mean values were normalized to the housekeeping genes β-Actin, RPLP0 or GAPDH. Gene expression analysis of 83 DNA repair genes was performed with the RT^2 Profiler PCR Array Human DNA Repair (Qiagen, #PAHS-042Z) according to the manufacturer's instructions. For RT^2 profiling, equal quantities of RNA from three independent repeats were used. Results for ATR, BRCA1 and BRCA2 were validated with qPCR of the independent repeats.

Protein isolation and Western Blot

For protein isolation, cells were washed twice with PBS and lysed with RIPA buffer (Fischer Scientific, Waltham, Massachusetts) supplemented with complete inhibitor (Roche), proteinase and phosphatase inhibitor (Fisher Scientific). Lysates were centrifuged for 10

minutes at 10,000 rpm, 4°C to remove cell debris. Afterwards, total protein concentration was determined using the Pierce™ BCA Protein Assay kit (Fisher Scientific) according to the manufacturer's instructions. Next, protein lysates were mixed with 4x loading buffer and heated to 95°C for five minutes. 10 µg or higher of total protein along with a molecular weight marker (Fisher Scientific) were loaded onto a 4-12 % gradient polyacrylamide gel (Invitrogen) and proteins were separated for approximately 90 minutes at 120 V in a Cell Sure Lock™ electrophoresis cell containing 1x NuPAGE™ running buffer (both Invitrogen). Next, proteins were blotted onto a Amersham™ Protran® 0.2 µm nitrocellulose membrane (Sigma-Aldrich) by wet transfer at 100 V for 90 minutes using NuPAGE™ transfer buffer (Fisher Scientific,) in a cooled Mini-PROTEAN®© 3 transfer tank (Bio-Rad). After transfer, membranes were washed with 1x PBS with 0.1% Triton X-100 (PBS-T) and blocked with 5 % BSA in PBS-T for 1 hour at room temperature. Next, incubation with the primary antibody was performed overnight at +4°C. Primary antibodies were diluted as 1:1000 in 5 % BSA in PBS-T. Secondary antibodies were diluted as 1:10000 in 5 % BSA in PBS-T. The list of primary and secondary antibodies can be found in Supplementary Table 2. GAPDH was used as housekeeping protein. On the next day, primary antibodies were removed and membranes were repeatedly washed with PBS-T for at least 90 minutes. Incubation with the secondary antibody was performed for one hour at room temperature. After additional washing with PBS-T for at least two hours, membranes were incubated with SuperSignal West Dura Extended Duration Substrate kit (Fischer Scientific) or HRP Detection Reagent (GE Healthcare). Chemiluminescent signals were detected using X-ray film (Agfa).

Cell cycle analysis

Analysis of cell cycle distribution was performed on HNSCC cells seeded at low cell density (10^5 cells/well in 6-well plate) to match the exponential growth phase. Harvested cells were washed in Flow Cytometry buffer (1x DPBS, 1 mM EDTA, 25 mM HEPES, 3 % FBS, all Sigma-Aldrich) and subsequently incubated with 10 µg/ml Hoechst 33342 DNA dye (Invitrogen) diluted in Flow Cytometry buffer. After 45 minutes incubation at 37°C, the

reaction was stopped by placing the samples on ice. Immediately prior to analysis, 2 µg/ml of the live-dead marker 7-Aminoactinomycin (7-AAD) (Sigma-Aldrich) was added and Flow Cytometry was conducted using the BD FACSCelesta™ Flow Cytometer (BD). The subsequent data analysis was performed with FlowJo™ v10 (FlowJo LLC).

γH2A.X staining

For staining of γH2A.X foci, cells were plated onto 8-well Millicell® EZ chamber slides (Merck Millipore) at a density of 25 000 cells/well. Next day, cells received 4 Gy X-ray irradiation delivered by Yxlon Y.TU 320 (200 kV X-rays, dose rate 1.3 Gy/minute at 20 mA, filtered with 0.5 mm Cu). Absorbed dose was measured using a Duplex dosimeter (PTW). Sham-irradiated cells were used as control. 24 h after irradiation, cells were fixed with 3.7% formaldehyde (Thermo Fisher Scientific) for 30 minutes at 37°C. After washing three times with PBS, the samples were permeabilized using 0.25% Triton X100 (Sigma-Aldrich) for seven minutes at room temperature and again washed with PBS. Next, samples were blocked with 10% BSA (Fisher Scientific) in PBS at 37°C for one hour. Incubation with the primary antibody diluted as 1:400 in 3% BSA/PBS was conducted overnight at +4°C. On the next day, samples were repeatedly washed with PBS and subsequently incubated with the secondary antibody diluted as 1:500 in 3% BSA/PBS for one hour at room temperature. After additional washing steps in PBS, nuclear staining was performed with 1 µg/ml DAPI for 5 minutes. The images were taken with a confocal Leica SP5 microscope. Images were analyzed using Fiji/ImageJ [13].

CRISPR/Cas9-mediated Oct4 A knockout

Oct4 A KO clones were generated from the UTSCC5 cell line using the CRISPR/Cas9 system. A single-guided (sg) RNA oligonucleotide duplex specifically targeting exon 1 in the Oct4 A isoform was designed with the CCTop-CRISPR/Cas9 target online predictor [15, 16], purchased from Eurofins Genomics and cloned into a pGuideIt vector plasmid (Takara Bio). Next, UTSCC5 cells were co-transfected with pGuideIt-Oct4-sgRNA plasmid and

pSpCas9(BB)-2A-GFP plasmid (gift from Feng Zhang, Addgene plasmid # 48138), which was additionally modified by including the high fidelity Cas9 enzyme Cas9-HF1 (gift from Aliona Bogdanova, Max Planck Institute of Molecular Cell Biology and Genetics). Then, transfected cells identified by GFP expression were sorted as single cells into the wells of a 96-well plate. After sorting, cells were grown in complete DMEM for several weeks. Clones were sequenced by Eurofins Genomics to detect the presence of frameshift insertion or deletion mutations in the targeted area. For further validation, absence of intact exon 1 was confirmed with PCR using indel-sensitive primers. Primer sequences are provided in Supplementary Table 2. The presence of intact copies of the non-targeted neighbor exon 2b was used as control.

Chromatin immunoprecipitation (ChIP)

ChIP experiments were conducted predominantly with the Chromatin Immunoprecipitation (ChIP) Assay Kit (Merck Millipore) according to the manufacturer`s instructions. Importantly, DNA fragmentation step was performed using Micrococcal Nuclease (Cell Signaling Technology) according to the ChIP protocol of the manufacturer. In brief, when NCCIT cells reached a density of $3x10^6$ cells per 10 cm^2 dish, proteins were crosslinked to the DNA by incubation with 1% formaldehyde (Thermo Fisher Scientific) for 15 minutes at 37°C. Cells were collected in PBS containing ptotease inhibitors (1 mM PMSF, Sigma-Aldrich), 1 µg/ml aprotinin, 1 µg/ml Pepstatin A, (both Biomol) and lysed in the recommended buffer from the Cell Signaling Technology protocol. Next, nuclear DNA was fragmented by incubating nuclei with 0.125 µl Micrococcal Nuclease (Cell Signaling Technology) for 10 minutes at 37°C. After that, nuclei were transferred to SDS Lysis buffer (Merck Millipore) and following steps were performed according to the Merck Millipore ChIP protocol. Disruption of nuclei was achieved by repeatedly passing the suspension through an insulin syringe. Then, samples were incubated with primary antibody against Oct4 (Abcam, Cell signaling technology) or control IgG antibody (Cell signaling technology) overnight at 4°C. The next day, DNA-protein-antibody complexes were precipitated using Agarose beads and crosslinks were reversed at

65°C for 4 h. The DNA fragments were further purified using the QIAquick PCR Purification Kit (Qiagen). For qPCR detection of immunoprecipitated DNA fragments, primers were designed to cover different promotor regions of the presumable Oct4 target genes which contained putative Oct4 binding sites (predicted by Eukaryotic Promotor Database, https://epd.epfl.ch//index.php). Significance of DNA fragment yield after Oct4 antibody precipitation compared to control IgG was calculated using paired *t*-test.

TCGA dataset analysis

For analysis of the Oct4-correlating gene signature, the TCGA HNSCC provisional dataset was used (519 patients, 119 radiotherapy-treated). Oct4-correlating genes were identified with Pearson correlation analysis in the SUMO software (https://angiogenesis.dkfz.de/oncoexpress/software/sumo/) (5). Kaplan-Meier survival curves were generated with log-rank test employing the optimal threshold method to define the high-expression and low-expression subgroup. For evaluation of target gene co-expression with DNA repair genes, median R values were determined using Pearson correlation analysis and statistical significance was calculated by Wilcoxon signed rank test of obtained R values against R=0 (no correlation). The subsequent pathway analysis was performed with the Reactome Pathway Database (https://reactome.org/). Gene expression differences between tumor and normal tissue were analyzed with the Gene Expression Profiling Interactive Analysis (GEPIA) web tool [17] based on TCGA and GTx data. P values < 0.01 were considered statistical significant.

Statistical analysis

The results of the sphere formation assay, yH2A.X assay and relative gene expression determined by qPCR were analyzed by paired two-tailed *t*-test. Statistical outliers were excluded based on Grubbs analysis (alpha 0.05). Sample sizes were generally determined based on the previous studies involving similar experimental setup. In general, at least three biological replicates of each experiment were performed.

Supplementary discussion

The correlation of Oct4 expression and HPV status

According to the previously published studies, the expression of Oct4 transcript might be induced by HPV16 oncoproteins suggesting a contribution of Oct4 to HPV–related tumorigenicity [5, 6]. The Oct4-mediated phenotype depends on the HPV status in cervical carcinoma: proliferation and migration were induced by Oct4 in HPV-negative cells whereas they were inhibited in HPV-positive cells [6]. Our finding indicates a significant correlation of the HPV status and low expression of Oct4 protein at the invasive front. Due to the high demand for new biomarkers, particularly for HPV-negative HNSCC, this report focuses on analyzing the role of Oct4 in the radioresistance of HPV-negative models.

Oct4 contributes to the maintenance of CSC phenotype

Allowing for efficient repair of irradiation-induced DNA double-strand breaks (DSB), an enhanced DNA damage response critically mediates CSC radioresistance via the activation of ATM and ATR kinase, cell cycle checkpoint regulators and DNA repair proteins [18, 19]. Consequently, an upregulation of the homologous recombination repair (HRR) pathway is associated with increased radioresistance in HNSCC cell lines [20], and HRR deficiencies are suggested as potential therapeutic targets [21]. Similarly, improved chemotherapy response was reported in patients with HNSCC with loss-of-function mutations in the DNA damage response gene *REVL13* [22]. HNSCC CSC populations can be identified using functional characteristics like low proteasome activity [23] and enhanced ATP-binding cassette (ABC) transporter activity [24]. Oct4 has been shown to regulate the CSC phenotype in different tumor entities [25-27]. In line with these reports, HNSCC cell lines treated with siRNA against Oct4 isoforms exhibited downregulation of the hyaluronic acid receptor CD44, an established HNSCC CSC biomarker that has been associated with decreased loco-regional control in patients with HNSCC after PORT-C or primary radiotherapy [28, 29]. Previous investigations suggested that CSC-related signaling in HNSCC cells depends on the interaction of the CD44v3 with Oct4 and other stemness transcription factors [30]. We found a significant alteration of the CSC-related gene signature

in Oct4 knockdown HNSCC cell lines. Oct4 downregulation also affected the functional characteristics of the CSC phenotype, as shown by decreased self-renewal capacity of Cal33 cells. The relevance of these *in vitro* findings for patients with HNSCC is supported by previous studies revealing an association of Oct4/CIP2A positivity with poor tumor differentiation status [31]. In addition to the effect on CSC characteristics, Cal33 cells exhibited partial radiosensitization upon Oct4 knockdown, whereas UTSCC5 cells were less affected regarding both self-renewal capacity and clonogenic survival after irradiation. In contrast to previous reports showing the different functions of Oct4 isoform A and B in stemness and stress response, neither downregulation of all Oct4 isoforms nor knockout of the stem cell-related isoform A led to significant radiosensitization of UTSCC5 cells, suggesting a cell line-specific effect [32-34]. Similar to the effect of Oct4 knockdown, overexpression of Oct4 in Cal33 cells also resulted in the accumulation of unrepaired DNA damage and significant cell radiosensitization. This finding is in line with previous observations that Oct4-dependent transcriptional regulation depends on its precise intracellular level, and both loss and gain of Oct4 expression led to the inhibition of the stem cell transcriptional program and ESC differentiation [35, 36].

CSCs of different tumor entities critically depend on the ATR/CHK1 signaling axis for an efficient DNA damage response, rendering them particularly sensitive towards CHK1 inhibition [37-39]. Upon irradiation, CHK1/WEE1-mediated G2 arrest constitutes a crucial survival mechanism for TP53 mutant cancers cells, which lack an adequate G1 checkpoint [40, 41]. With its various functions in the DNA damage response, TP53 mutations are considered an important driver of HPV-negative HNSCC carcinogenesis, occurring in more than 70% of cases [42, 43]. In HPV-positive tumors, the function of TP53 is antagonized by the viral oncoprotein E6 [44]. Of note, all cell lines used in this report possess TP53 mutations, highlighting the importance of alternative cell cycle checkpoints in our model systems. Our data demonstrated that Oct4 knockdown in UTSCC5 cells abrogates the irradiation-induced G2-arrest, which could be partially attributed to the downregulation of the Oct4-driven *CHEK-1* and *WEE1* expression.

Therefore, the results of our study demonstrated that Oct4 contributes to radioresistance of HNSCC by regulation of the CSC properties.

Oct4 A knockout cells activate pro-survival mechanisms

Our studies showed that Oct4 A knockout cells exhibit increased ATR expression compared to wild type cells, but failed to upregulate ATR expression upon irradiation. In addition, a trend towards higher CHK1 protein levels and increased phosphorylation was observed in Oct4 A knockout cells. As CHK1 phosphorylation is a well-established effect of activated ATR signaling [2], our findings suggest that during long-term culturing, Oct4 A knockout clones potentially acquired additional changes leading to the activation of pro-survival mechanisms.

Supplementary Figures and Tables

Supplementary Table 1: Oct4 expression at the invasive front and HPV16 status crosstabulation

Supplementary Table 2: Antibodies, primers and siRNA oligonucleotides used for the study

Supplementary Figure 1. Analysis of the Oct4 expression in HNSCC cells. (A) Schematic representation of the different Oct4 transcript variants (black) and protein isoforms (blue) encoded by the *POU5F1* gene. (B) Western blot analysis in Cal33, UTSCC5, FaDu and FaDu radioresistant (RR) HNSCC cell lines and diluted samples for NCC-IT cells with and without transfection with pooled Oct4 A siRNA 1 and 2. Cells transfected with scrambled (Scr) siRNA were used as control. Proteins were detected using anti-Oct4 (Cell Signaling Technology, CST #2750) antibodies. (C) Quantitative real-time PCR (RT-qPCR) analysis of Oct4 A and Oct4 B expression in Cal33 and UTSCC5 cell lines after siRNA-mediated knockdown of Oct4 A expression; error bars indicate SD; *- $p < 0,05$. Cells after Oct4 A knockdown were analyzed in the sphere forming assay depicted in Figure 1C, RT-qPCR depicted in Figure 1B and cell cycle analysis depicted in Figure 1E. (D) Expression of

ALDH1A1 and *ALDH1A3* genes in HNSCC cells after Oct4 knockdown; error bars indicate SD; *- p < 0,05.

Supplementary Figure 2. Oct4 regulates *CHEK1* and *WEE1* expression. (A) Oct4 knockdown in HNSCC cells resulted in a downregulation of *CHEK1* and *WEE1* expression; error bars indicate SD; *- p < 0,05. (B) Western blot analysis of Chk1 expression in Cal33, UTSCC5, FaDu and FaDu radioresistant (RR) HNSCC cell with and without transfection with pooled Oct4 A siRNA 1 and 2. Cells transfected with scrambled (Scr) siRNA were used as control. Western blot analysis for Oct4 protein is shown in Supplementary Figure 1B. Western blot analysis for GAPDH is the same as in Supplementary Figure 1B. (C) Analysis of the *CHEK1* and *WEE1* gene promoters using The Eukaryotic Promoter Database (EPD) revealed putative Oct4 binding elements.

Supplementary Figure 3. Plating efficacy of Cal33 (A) and UTSCC5 (B) after siRNA-mediated knockdown of Oct4 A and Oct4 B expression. Error bars indicate SD. Cells after Oct4 A or Oct4 B knockdown were analyzed by the radiobiological colony forming assay depicted in Figure 2B.

Supplementary Figure 4. Characterization of HNSCC cells with deregulated Oct4 expression levels. (A) Schematic representation of the CRISPR/Cas9 system which has been used for Oct4 knockout (KO) generation in UT SCC5 cells. The candidate clones were checked for mutations by sequencing and validated for the absence of intact copies of Oct4A via PCR with indel sensitive primers. A PCR for non-targeted neighbor exon 2b was used as control. MaKo – monoallelic knockout with in-frame indel mutation of second allele. Wild type (WT) clones underwent the same procedure but did not have mutations in Oct4 DNA sequence. (B) Quantitative real-time PCR (RT-qPCR) analysis of Oct4 A, Oct4 B and OCT4 B1 expression in UTSCC5 cell lines after introduction of the CRISPR/Cas9 – mediated frameshift mutations in Oct4 KO clones; error bars indicate SD; *- p < 0,05. (C) Plating

efficacy of WT and Oct4 KO UTSCC5 cells in 2D and 3D Matrigel-based cell culture conditions; error bars indicate SD. WT and Oct4 KO UTSCC5 cells were analyzed by the radiobiological colony forming assays depicted in Figure 2C. (D) Plating efficacy of Cal33 cells stably transfected with pWPXL-tdTomato or pWPXL-Oct4-HA-tdTomato plasmids. These cells were analyzed by the radiobiological colony forming assay depicted in Figure 2D; error bars indicate SD. (E) Relative mRNA expression levels in Cal33 pWPXL-Oct4-HA-tdTomato cells relatively to Cal33 pWPXL-tdTomato cells; error bars indicate SD; *- p < 0,05.

Supplementary Figure 5. The impact of Oct4 expression on HNSCC tumor radioresistance and on the properties of UTSCC5 cells with CRISPR/Cas9 mediated Oct4 knockout.

(A) Kaplan-Meier analysis of patients with HNSCC treated with postoperative radio(chemo)therapy (PORT-C). The impact of Oct4 expression on loco-regionalloco-regional control was evaluated using the univariate Cox-regression model. Statistical analysis was performed by SPSS software. High (intensity 2) and low (intensity 0) nuclear Oct4 expression at the invasive front is associated with better loco-regional control; n = 167. (B) Plating efficacy of WT and Oct4 KO UTSCC5 cells pretreated with Olaparib at a concentration of 1µM for 2h before irradiation. Untreated cell were used as control; error bars indicate SD; **- p < 0,01, *** - p < 0,001, **** - p < 0,0001. Cells were analyzed by the radiobiological colony forming assays depicted in Figure 3A. (C) Expression of *ATR, BRCA1* and *BRCA2* genes in UTSCC5 Oct4 A KO and WT clones; error bars indicate SD; *- p < 0,05. (D) Western blot analysis of Chk1 and phospho-Chk1 (S296) expression in Oct4 KO and WT cells; error bars indicate SD.

Supplementary Figure 6. Association of mRNA expression of *PSMC3IP* and *RAD54L* genes with overall survival probability in HNSCC patients. Association of mRNA expression of *PSMC3IP* and *RAD54L* with overall survival probability in the total HNSCC patient cohort, n = 517 (A) and in the cohort of the HNSCC patients treated with

radiotherapy, n = 119 (B). Small variances in the patients' numbers compared to Figure 4 are due to different version of TCGA HNSCC dataset used by the different web tools.

Supplementary Figure 7. Analysis of the signaling pathways linked to *PSMC3IP*, *RAD54L* and *CHEK1*. (A) Co-expression analysis of *PSMC3IP* and *RAD54L* with *CHEK1* using HNSCC patient cohort identified the clusters of highly correlating genes. The cluster of genes highly correlating with *PSMC3IP* and *CHEK1* (B) or with *RAD54L* and *CHEK1* (C) were used for pathway analysis using the Reactome Pathway Database (https://reactome.org/).

Supplementary Figure 8. Analysis of the Oct4-depedend regulation of the putative target genes. (A) Analysis of the promoter regions of *PSMC3IP* and *RAD54L* genes using The Eukaryotic Promoter Database (EPD) revealed potential Oct4 binding sites. (B) qRT-PCR analysis of the gene expression levels of Oct4A and putative Oct4 target genes PSMC3IP, RAD54L and CHEK1 in NCC-IT pluripotent embryonal carcinoma cells; error bars indicate SD. (C) qRT-PCR time-course analysis of CHEK1, PSMC3IP, and RAD54L expression in Oct4 siRNA transfected Cal33 cells; error bars indicate SD; *- p < 0,05; **- p < 0,01.

Supplementary Figure 9. Correlation of *POU5F1*, *RAD54L* and *PSMC3IP* expression with genomic instability and HPV p16 status in HNSCC patients. (A) Correlation of fraction genome altered (FGA) index with mRNA expression of PSMC3IP, RAD54L, and POU5F1 in the TCGA HNSCC patient cohort, n = 519. (B) Increased expression levels of POU5F1, PSMC3IP, and RAD54L in HPV16-positive tumors versus HPV16-negative tumors in the TCGA HNSCC patient cohort. (C) Association of low expression of DNA repair gene signature with worse survival in HPV16-negative HNSCC patients. PSMC3IP and RAD54L genes are highlighted in blue.

Supplementary Figure 10. Quantitative real-time PCR (RT-qPCR) analysis of *PSMC3IP* and *RAD54L* expression in Cal33 and UTSCC5 cell lines after siRNA-mediated knockdown. Error bars indicate SD; *- p < 0,05. Cells after *PSMC3IP* and *RAD54L* knockdown were analyzed by the sphere forming assay depicted in Figure 5D.

Supplementary Figure 11. Plating efficiency of Cal33 and UTSCC5 cells after siRNA-mediated knockdown of *PSMC3IP* and *RAD54L* expression. Error bars indicate SD. Cells after *PSMC3IP* and *RAD54L* knockdown were analyzed by the radiobiological colony forming assay depicted in Figure 6B.

References:

1 Villodre ES, Kipper FC, Pereira MB, Lenz G. Roles of OCT4 in tumorigenesis, cancer therapy resistance and prognosis. *Cancer Treat Rev* 2016; 51: 1-9.

2 Zhao H, Piwnica-Worms H. ATR-mediated checkpoint pathways regulate phosphorylation and activation of human Chk1. *Mol Cell Biol* 2001; 21: 4129-4139.

3 Akagi K, Li J, Broutian TR, Padilla-Nash H, Xiao W, Jiang B *et al*. Genome-wide analysis of HPV integration in human cancers reveals recurrent, focal genomic instability. *Genome Res* 2014; 24: 185-199.

4 Korzeniewski N, Spardy N, Duensing A, Duensing S. Genomic instability and cancer: lessons learned from human papillomaviruses. *Cancer Lett* 2011; 305: 113-122.

5 Liu D, Zhou P, Zhang L, Wu G, Zheng Y, He F. Differential expression of Oct4 in HPV-positive and HPV-negative cervical cancer cells is not regulated by DNA methyltransferase 3A. *Tumour Biol* 2011; 32: 941-950.

6 Panayiotou T, Michael S, Zaravinos A, Demirag E, Achilleos C, Strati K. Human papillomavirus E7 binds Oct4 and regulates its activity in HPV-associated cervical cancers. *PLoS Pathog* 2020; 16: e1008468.

7 Kang J, D'Andrea AD, Kozono D. A DNA repair pathway-focused score for prediction of outcomes in ovarian cancer treated with platinum-based chemotherapy. *J Natl Cancer Inst* 2012; 104: 670-681.

8 Pitroda SP, Pashtan IM, Logan HL, Budke B, Darga TE, Weichselbaum RR *et al*. DNA repair pathway gene expression score correlates with repair proficiency and tumor sensitivity to chemotherapy. *Sci Transl Med* 2014; 6: 229ra242.

9 Ciccia A, Elledge SJ. The DNA damage response: making it safe to play with knives. *Mol Cell* 2010; 40: 179-204.

10 Digomann D, Kurth I, Tyutyunnykova A, Chen O, Lock S, Gorodetska I *et al*. The CD98 Heavy Chain Is a Marker and Regulator of Head and Neck Squamous Cell Carcinoma Radiosensitivity. *Clin Cancer Res* 2019; 25: 3152-3163.

11 Lohaus F, Linge A, Tinhofer I, Budach V, Gkika E, Stuschke M *et al*. HPV16 DNA status is a strong prognosticator of loco-regional control after postoperative radiochemotherapy of locally advanced oropharyngeal carcinoma: results from a multicentre explorative study of the German Cancer Consortium Radiation Oncology Group (DKTK-ROG). *Radiother Oncol* 2014; 113: 317-323.

12 Franken NA, Rodermond HM, Stap J, Haveman J, van Bree C. Clonogenic assay of cells in vitro. *Nat Protoc* 2006; 1: 2315-2319.

13 Schindelin J, Arganda-Carreras I, Frise E, Kaynig V, Longair M, Pietzsch T *et al*. Fiji: an open-source platform for biological-image analysis. *Nat Methods* 2012; 9: 676-682.

14 Peitzsch C, Cojoc M, Hein L, Kurth I, Mabert K, Trautmann F *et al*. An Epigenetic Reprogramming Strategy to Resensitize Radioresistant Prostate Cancer Cells. *Cancer research* 2016; 76: 2637-2651.

15 Labuhn M, Adams FF, Ng M, Knoess S, Schambach A, Charpentier EM *et al*. Refined sgRNA efficacy prediction improves large- and small-scale CRISPR-Cas9 applications. *Nucleic Acids Res* 2018; 46: 1375-1385.

16 Stemmer M, Thumberger T, Del Sol Keyer M, Wittbrodt J, Mateo JL. CCTop: An Intuitive, Flexible and Reliable CRISPR/Cas9 Target Prediction Tool. *PLoS One* 2015; 10: e0124633.

17 Tang Z, Li C, Kang B, Gao G, Li C, Zhang Z. GEPIA: a web server for cancer and normal gene expression profiling and interactive analyses. *Nucleic Acids Res* 2017; 45: W98-W102.

18 Schulz A, Meyer F, Dubrovska A, Borgmann K. Cancer Stem Cells and Radioresistance: DNA Repair and Beyond. *Cancers (Basel)* 2019; 11.

19 Krause M, Dubrovska A, Linge A, Baumann M. Cancer stem cells: Radioresistance, prediction of radiotherapy outcome and specific targets for combined treatments. *Adv Drug Deliv Rev* 2017; 109: 63-73.

20 Liu C, Liao K, Gross N, Wang Z, Li G, Zuo W *et al*. Homologous recombination enhances radioresistance in hypopharyngeal cancer cell line by targeting DNA damage response. *Oral Oncol* 2020; 100: 104469.

21 Wurster S, Hennes F, Parplys AC, Seelbach JI, Mansour WY, Zielinski A *et al*. PARP1 inhibition radiosensitizes HNSCC cells deficient in homologous recombination by disabling the DNA replication fork elongation response. *Oncotarget* 2016; 7: 9732-9741.

22 Huang KK, Jang KW, Kim S, Kim HS, Kim SM, Kwon HJ *et al*. Exome sequencing reveals recurrent REV3L mutations in cisplatin-resistant squamous cell carcinoma of head and neck. *Sci Rep* 2016; 6: 19552.

23 Lagadec C, Vlashi E, Bhuta S, Lai C, Mischel P, Werner M *et al*. Tumor cells with low proteasome subunit expression predict overall survival in head and neck cancer patients. *BMC Cancer* 2014; 14: 152.

24 Song J, Chang I, Chen Z, Kang M, Wang CY. Characterization of side populations in HNSCC: highly invasive, chemoresistant and abnormal Wnt signaling. *PLoS One* 2010; 5: e11456.

25 Kumar SM, Liu S, Lu H, Zhang H, Zhang PJ, Gimotty PA *et al*. Acquired cancer stem cell phenotypes through Oct4-mediated dedifferentiation. *Oncogene* 2012; 31: 4898-4911.

26 Manic G, Signore M, Sistigu A, Russo G, Corradi F, Siteni S *et al*. CHK1-targeted therapy to deplete DNA replication-stressed, p53-deficient, hyperdiploid colorectal cancer stem cells. *Gut* 2018; 67: 903-917.

27 Song B, Kim DK, Shin J, Bae SH, Kim HY, Won B *et al*. OCT4 directly regulates stemness and extracellular matrix-related genes in human germ cell tumours. *Biochem Biophys Res Commun* 2018; 503: 1980-1986.

28 de Jong MC, Pramana J, van der Wal JE, Lacko M, Peutz-Kootstra CJ, de Jong JM *et al*. CD44 expression predicts local recurrence after radiotherapy in larynx cancer. *Clin Cancer Res* 2010; 16: 5329-5338.

29 Linge A, Lohaus F, Lock S, Nowak A, Gudziol V, Valentini C *et al*. HPV status, cancer stem cell marker expression, hypoxia gene signatures and tumour volume identify good prognosis subgroups in patients with HNSCC after primary radiochemotherapy: A multicentre retrospective study of the German Cancer Consortium Radiation Oncology Group (DKTK-ROG). *Radiother Oncol* 2016; 121: 364-373.

30 Bourguignon LY, Wong G, Earle C, Chen L. Hyaluronan-CD44v3 interaction with Oct4-Sox2-Nanog promotes miR-302 expression leading to self-renewal, clonal formation, and cisplatin resistance in cancer stem cells from head and neck squamous cell carcinoma. *J Biol Chem* 2012; 287: 32800-32824.

31 Ventela S, Sittig E, Mannermaa L, Makela JA, Kulmala J, Loyttyniemi E *et al*. CIP2A is an Oct4 target gene involved in head and neck squamous cell cancer oncogenicity and radioresistance. *Oncotarget* 2015; 6: 144-158.

32 Gao Y, Wei J, Han J, Wang X, Su G, Zhao Y *et al*. The novel function of OCT4B isoform-265 in genotoxic stress. *Stem Cells* 2012; 30: 665-672.

33 Wang X, Zhao Y, Xiao Z, Chen B, Wei Z, Wang B *et al*. Alternative translation of OCT4 by an internal ribosome entry site and its novel function in stress response. *Stem Cells* 2009; 27: 1265-1275.

34 Xiao M, Liu L, Zhang S, Yang X, Wang Y. Cancer stem cell biomarkers for head and neck squamous cell carcinoma: A bioinformatic analysis. *Oncol Rep* 2018; 40: 3843-3851.

35 Hammachi F, Morrison GM, Sharov AA, Livigni A, Narayan S, Papapetrou EP *et al*. Transcriptional activation by Oct4 is sufficient for the maintenance and induction of pluripotency. *Cell Rep* 2012; 1: 99-109.

36 Niwa H, Miyazaki J, Smith AG. Quantitative expression of Oct-3/4 defines differentiation, dedifferentiation or self-renewal of ES cells. *Nat Genet* 2000; 24: 372-376.

37 Bartucci M, Svensson S, Romania P, Dattilo R, Patrizii M, Signore M *et al*. Therapeutic targeting of Chk1 in NSCLC stem cells during chemotherapy. *Cell Death Differ* 2012; 19: 768-778.

38 Meng L, Hu H, Zhi H, Liu Y, Shi F, Zhang L *et al*. OCT4B regulates p53 and p16 pathway genes to prevent apoptosis of breast cancer cells. *Oncol Lett* 2018; 16: 522-528.

39 Venkatesha VA, Parsels LA, Parsels JD, Zhao L, Zabludoff SD, Simeone DM *et al*. Sensitization of pancreatic cancer stem cells to gemcitabine by Chk1 inhibition. *Neoplasia* 2012; 14: 519-525.

40 Bridges KA, Hirai H, Buser CA, Brooks C, Liu H, Buchholz TA *et al*. MK-1775, a novel Wee1 kinase inhibitor, radiosensitizes p53-defective human tumor cells. *Clin Cancer Res* 2011; 17: 5638-5648.

41 Koniaras K, Cuddihy AR, Christopoulos H, Hogg A, O'Connell MJ. Inhibition of Chk1-dependent G2 DNA damage checkpoint radiosensitizes p53 mutant human cells. *Oncogene* 2001; 20: 7453-7463.

42 Zhou G, Liu Z, Myers JN. TP53 Mutations in Head and Neck Squamous Cell Carcinoma and Their Impact on Disease Progression and Treatment Response. *J Cell Biochem* 2016; 117: 2682-2692.

43 Leemans CR, Snijders PJF, Brakenhoff RH. The molecular landscape of head and neck cancer. *Nat Rev Cancer* 2018; 18: 269-282.

44 Leemans CR, Braakhuis BJ, Brakenhoff RH. The molecular biology of head and neck cancer. *Nat Rev Cancer* 2011; 11: 9-22.
