# Supplementary material for: Oct4 confers stemness and radioresistance to head and neck squamous cell carcinoma by regulating the homologous recombination factors PSMC3IP and RAD54L
Source: Oncogene. 2021 Jun 2;40(24):4214–28. doi: 10.1038/s41388-021-01842-1 (PMC8211562; doi:10.1038/s41388-021-01842-1)
Supplement: Supplementary file 2 — Supplementary Table 1 [file 41388_2021_1842_MOESM2_ESM.docx]

**Supplementary Table 1: Oct4 expression at the invasive front and HPV16 status crosstabulation**

| HPV16 status  Oct4 expression  at the invasive front | | 0 | 1 | Altogether |
| --- | --- | --- | --- | --- |
|  | 0 | 44 | 18 | 62 |
|  | 1 | 94 | 10 | 104 |
| Altogether | | 138 | 28 | 166 |

Chi square p-value = 0.001
