# Supplementary material for: Oct4 confers stemness and radioresistance to head and neck squamous cell carcinoma by regulating the homologous recombination factors PSMC3IP and RAD54L
Source: Oncogene. 2021 Jun 2;40(24):4214–28. doi: 10.1038/s41388-021-01842-1 (PMC8211562; doi:10.1038/s41388-021-01842-1)
Supplement: Supplementary file 3 — Supplementary Table 2 [file 41388_2021_1842_MOESM3_ESM.docx]

**Supplementary table 2: Antibodies, primers and siRNA oligonucleotides**

| **Antibodies** | | | | |
| --- | --- | --- | --- | --- |
| **Antigen** | **Company** | **Catalog #** | **Application** | |
| γH2A.X (p Ser139) | Novus Biologicals (Centennial, USA) | NB100-384 | γH2A.X foci assay | |
| RAD54L | Abcam (Cambridge, UK) | ab11055 | Western Blot | |
| PSMC3IP | Proteintech (Chicago, USA) | 11339-1-AP | Western Blot | |
| pChk1 (Ser296) | Cell Signaling Technology (Danvers, USA) | 2349 | Western Blot | |
| Oct4 | Abcam (Cambridge, UK) | ab19857 | Western Blot, Chromatin immunoprecipitation | |
| Oct4 | Abcam (Cambridge, UK) | ab18976 | Immunohistochemical tissue staining | |
| Oct4 | Cell Signaling Technology | 2750 | Chromatin immunoprecipitation | |
| GAPDH | Cell Signaling Technology | 2118 | Western Blot | |
| **Primers (qRT-PCR)** | | | | |
| **Primers** | **Product size (bp)** | **Sequences** | | **Tm, °C** |
| Oct4 isoform A | 224 | F 5`- CCGGAGCCCTGCACCGTCA-3`  R 5`-CGGCAGATGGTCGTTTGGCTGAAT- 3` | | 65,3  64,4 |
| Oct4 isoform B | 168 | F 5`- GTTCTTACAAGTCTTCTGCC -3`  R 5`- GCTGAATACCTTCCCAAATAG- 3` | | 55,3  55,9 |
| Oct4 isoform B1 | 192 | F 5`- CGTGCTCCCTCACTTTGCTTC-3`  R 5`- GGTTTCTGCTTTGCATATCTCCTG- 3` | | 61,8  61 |
| PSMC3IP | 149 | F 5`- CAGAAGAGAAAGAGCAGGTGTAC-3`  R 5-CTCTATCCCAACTTCCTCAAAGAAC- 3` | | 60,6  61,3 |
| RAD54L | 209 | F 5`- TTGAGTCAGCTAACCAATCAACC-3`  R 5`-GGAGGCTCATACAGAACCAAGG- 3` | | 58,9  62,1 |
| Chk1 | 184 | F 5`-CCAGATGCTCAGAGATTCTTCCA-3`  R 5`-TGTTCAACAAACGCTCACGATTA- 3` | | 60,6  57,1 |
| WEE1 | 205 | F 5`-GCAGAGTTGAAGGATCTCC-3`  R 5`-TTGTTACATGCCCAAGATCAC- 3` | | 56,7  55,9 |
| ATR | 165 | F 5`-GGCCAAAGGCAGTTGTATTGA-3`  R 5`-GTGAGTACCCCAAAAATAGCAGG- 3` | | 57.9  60,6 |
| BRCA1 | 183 | F 5`-CATCATTCACCCTTGGCACA-3`  R 5`-GGGGTATCAGGTAGGTGTCC- 3` | | 57,3  61,4 |
| BRCA2 | 227 | F 5`-CCACCACCACACAGAATTCT-3`  R 5`-ATGCAATAAACCTGAATCAGC- 3` | | 57,3  54 |
| GAPDH | 160 | F 5`- CAATGACCCCTTCATTGACC-3`  R 5`- TTGATTTTGGAGGGATCTCG - 3` | | 57,3  55,3 |
| RPLP0 | 177 | F 5`-CTCAACATCTCCCCCTTCTCCTT-3`  R 5`-TGATGCAACAGTTGGGTAGCC- 3` | | 62,4  59,8 |
| Β-Actin | 208 | F 5`- ATGGAGTCCTGTGGCATCCA-3`  R 5`- AGTACTTGCGCTCAGGAGGA - 3` | | 59,4  59,4 |
| **Primers (qPCR for chromatin immunoprecipitation)** | | | | |
| **Primers** | **Product size (bp)** | **Sequences** | | **Tm, °C** |
| NANOG | 160 | F 5`- GTTGCTGGGTTTGTCTTCAGG -3`  R 5`- GCGAGCACACACCCTACTG - 3` | | 59,8  61,0 |
| PSMC3IP region #1 | 178 | F 5`- GGTGCGAAGCTCGGTATTAGG -3`  R 5`- GTCACCAGAGCCCCGGACATCG - 3` | | 61,8  67,7 |
| PSMC3IP region #2 | 266 | F 5`- CGATGTCCGGGGCTCTGGTGAC -3`  R 5`- GATAGTGGTAGGAAAGTGGTTGTG - 3` | | 67,7  61,0 |
| PSMC3IP region #3 | 204 | F 5`- CACAACCACTTTCCTACCACTATC -3`  R 5`- GCAGCTGAGCACTGGTGACC - 3` | | 61,0  63,5 |
| RAD54L region #1 | 218 | F 5`- GTGAGTACCTAGGTTGTGC -3`  R 5`- GGTGTCTTGAAAGAAGCTG - 3` | | 56,7  54,5 |
| RAD54L region #2 | 229 | F 5`- CAGCTTCTTTCAAGACACC -3`  R 5`- CACCTGTAGTGGACAGCTG - 3` | | 54,5  58,8 |
| CHEK-1 region #1 | 149 | F 5`- CCAGTGCTGGAGAATGTAATGG -3`  R 5`- CTGCGTACGTCATTTAAGTGG - 3` | | 60,3  57,9 |
| CHEK-1 region #2 | 214 | F 5`- CCACTTAAATGACGTACGCAG -3`  R 5`- GTTGGAGCTTGAAGTCAGG - 3` | | 57,9  56,7 |
| CHEK-1 region #3 | 219 | F 5`- GTCCTTAAATCTCTTCAGCC -3`  R 5`- CTGCAGGAATCCAAATGCAG - 3` | | 55,3  57,3 |
| **Primers (PCR for validation of CRISPR/Cas9-mediated Oct4 A knockout)** | | | | |
| Oct4 Exon 1 | 207 | F 5`- GATGGCGTACTGTGGGACCC -3`  R 5`- CGTCGAAGCTCACTTGCCTC- 3` | | 63,5  61,4 |
| Oct4 Exon 2b | 163 | F 5`- CTGCCTTTTAAAATCCAGTCC-3`  R 5`-CAGAATCTGCAGAGGGGAAC-3` | | 55,9  59,4 |
| **siRNA oligonucleotides** | | | | |
| **Gene** | **siRNA name** | **Target sequence 5`-3`** | | |
| Control | Scrambled (Scr) siRNA | GCAGCUAUAUGAAUGUUGU | | |
| Oct4 isoform A | Oct4 A siRNA #1 | AGGAGAAGCUGGAGCAAAA | | |
| Oct4 isoform A | Oct4 A siRNA #2 | GAGUCGGGGUGGAGAGCAA | | |
| Oct4 isoform B | Oct4 B siRNA #1 | GAACUUAGCAGCUUAUCUA | | |
| Oct4 isoform B | Oct4 B siRNA #2 | GCUUAUAGAAGGUGCUCGA | | |
| Oct4 (total) | Oct4 siRNA | GUCCGAGUGUGGUUCUGUA | | |
| PSMC3IP | PSMC3IP-05 | ACAGAGAGAGGCAGAAGUA | | |
| PSMC3IP | PSMC3IP-07 | GCAACAAGGCAAGAUCAAA | | |
| PSMC3IP | PSMC3IP-08 | GAGAGAGAUUGAAGAACAU | | |
| RAD54L | RAD54L-05 | AGAAUGAUCUGCUUGAGUA | | |
| RAD54L | RAD54L-06 | CGAAUUACACCCAGACUUU | | |
| RAD54L | RAD54L-07 | GCACGAUGUCCAUUAAGAA | | |
| RAD54L | RAD54L-08 | AUACGGAGGACUUCUGAUA | | |
| Control  (for total Oct4 knockdown) | Scrambled (Scr) siRNA | AGGUAGUGUAAUCGCCUUG | | |
