# Supplementary figures and images for: Oct4 confers stemness and radioresistance to head and neck squamous cell carcinoma by regulating the homologous recombination factors PSMC3IP and RAD54L
Source: Oncogene. 2021 Jun 2;40(24):4214–28. doi: 10.1038/s41388-021-01842-1 (PMC8211562; doi:10.1038/s41388-021-01842-1)

Supplementary Figure 1

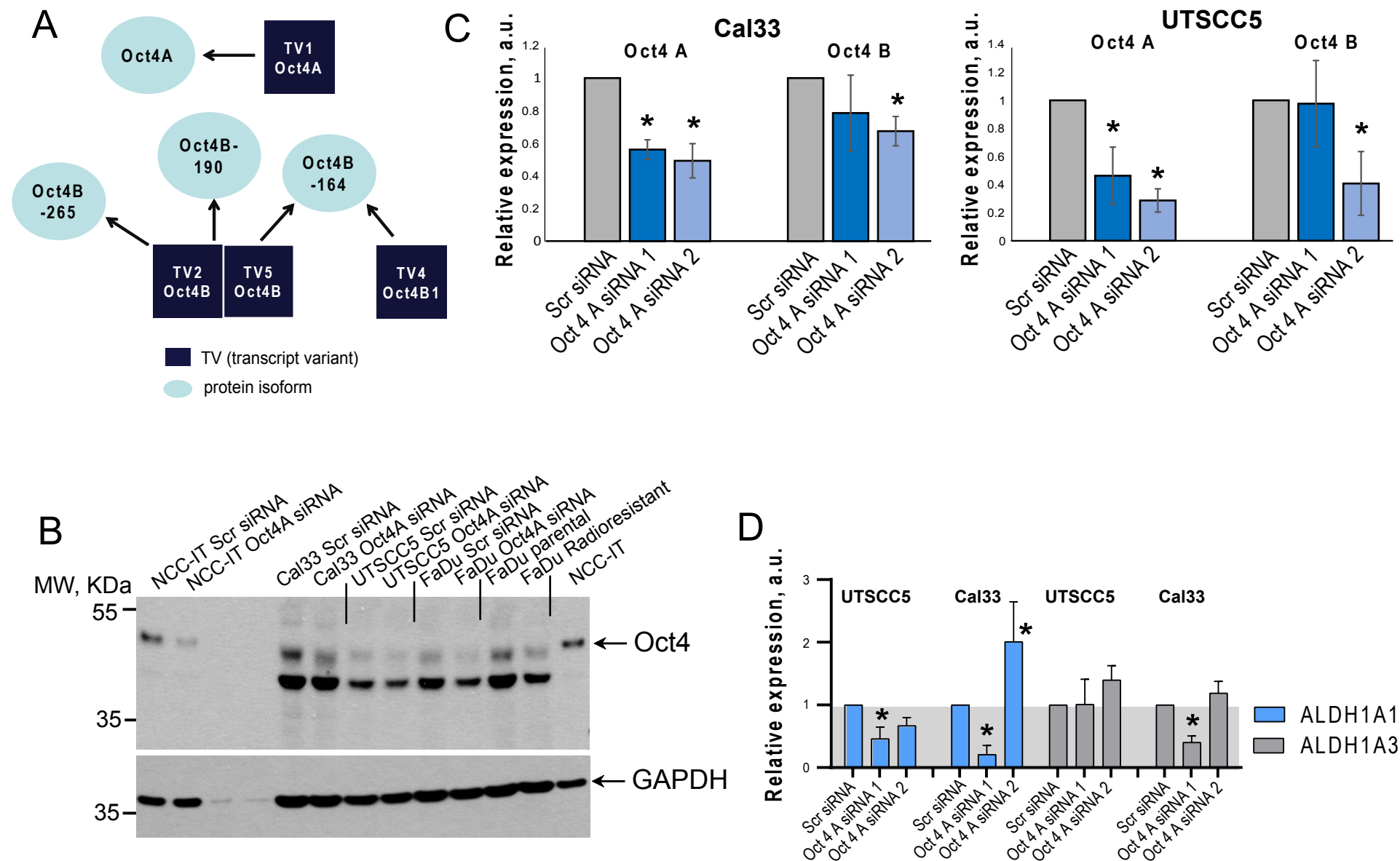

Supplement: Supplementary file 4 — Supplementary Figure 1 [file 41388_2021_1842_MOESM4_ESM.pdf]

Supplementary Figure 2

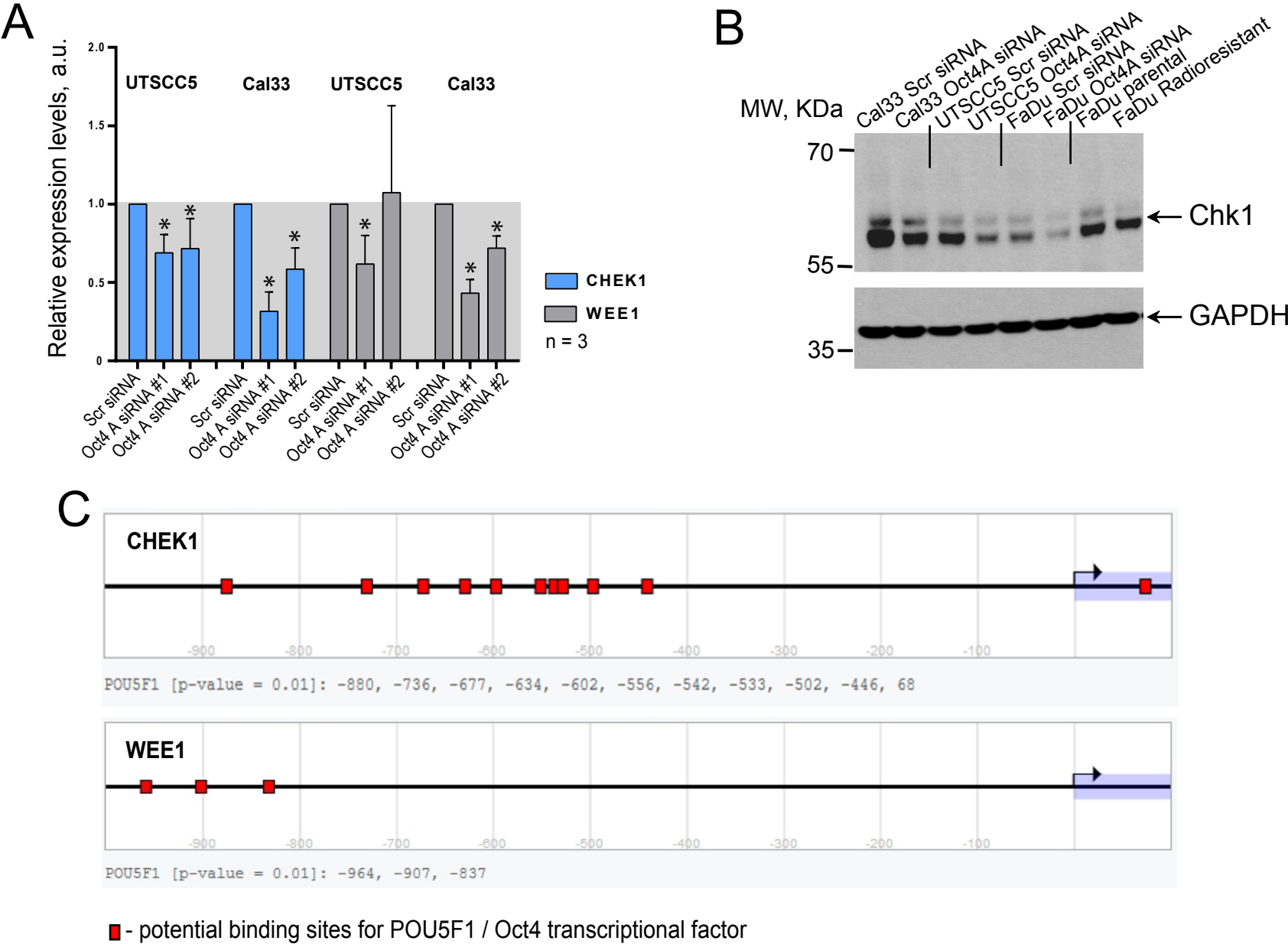

Supplement: Supplementary file 5 — Supplementary Figure 2 [file 41388_2021_1842_MOESM5_ESM.pdf]

Supplementary Figure 3

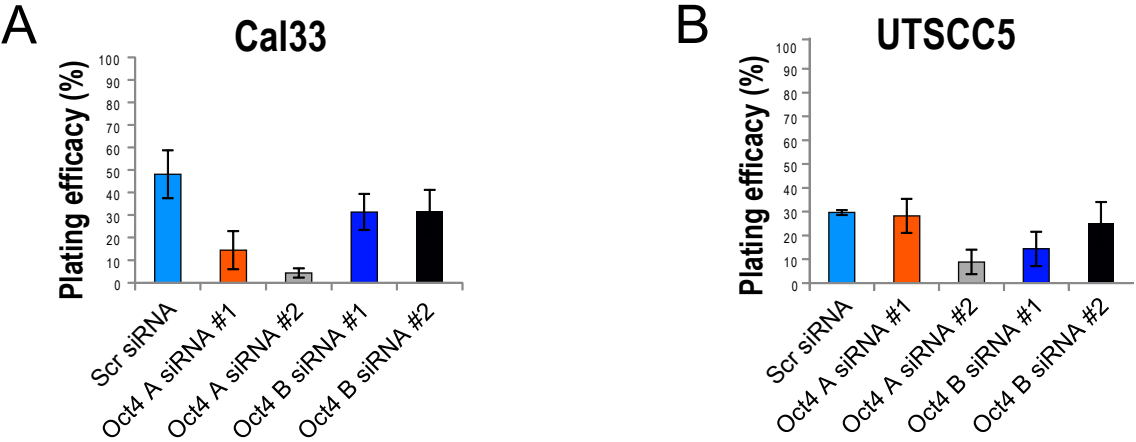

Supplement: Supplementary file 6 — Supplementary Figure 3 [file 41388_2021_1842_MOESM6_ESM.pdf]

## Supplementary Figure 4

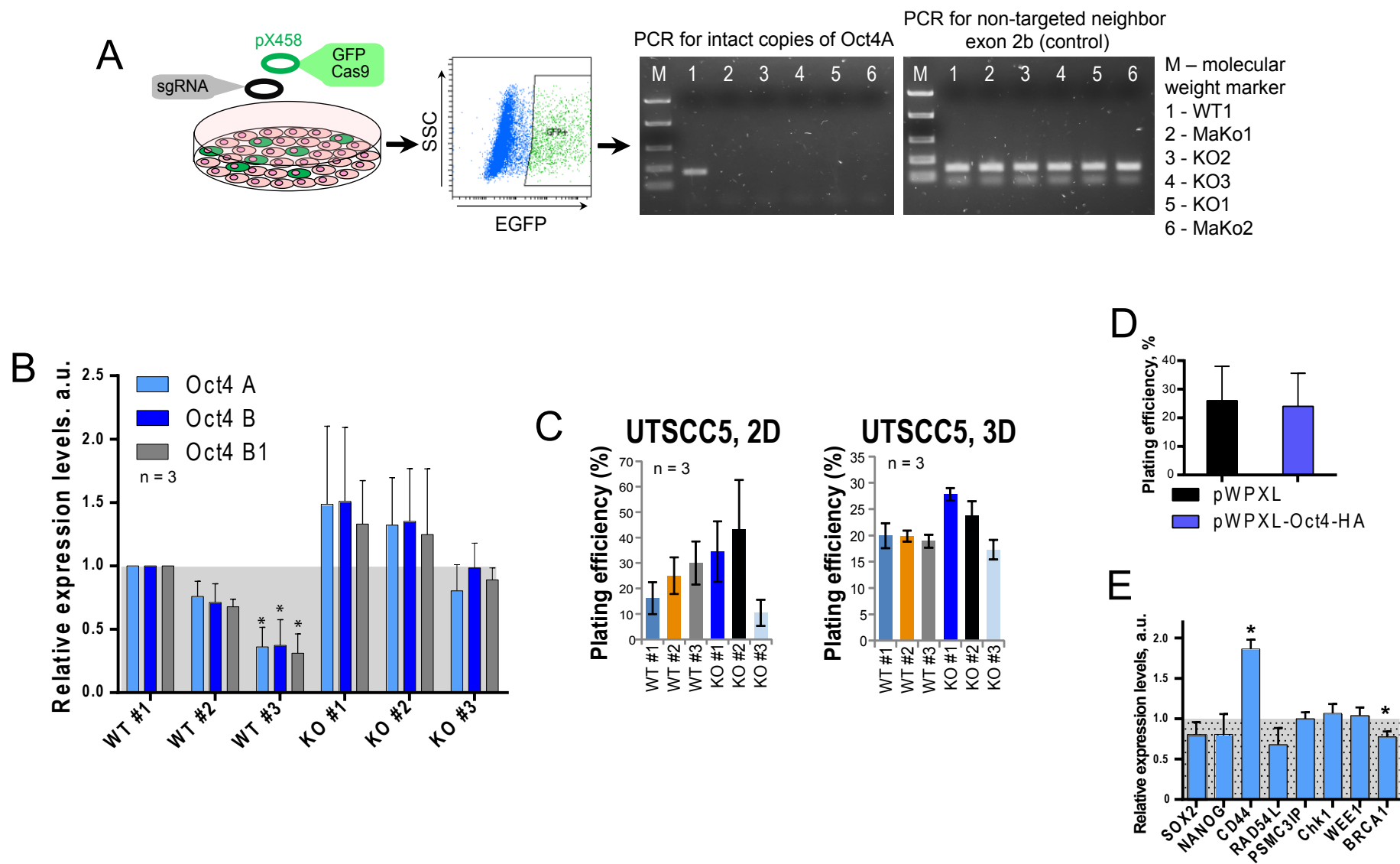

Supplement: Supplementary file 7 — Supplementary Figure 4 [file 41388_2021_1842_MOESM7_ESM.pdf]

Supplementary Figure 5

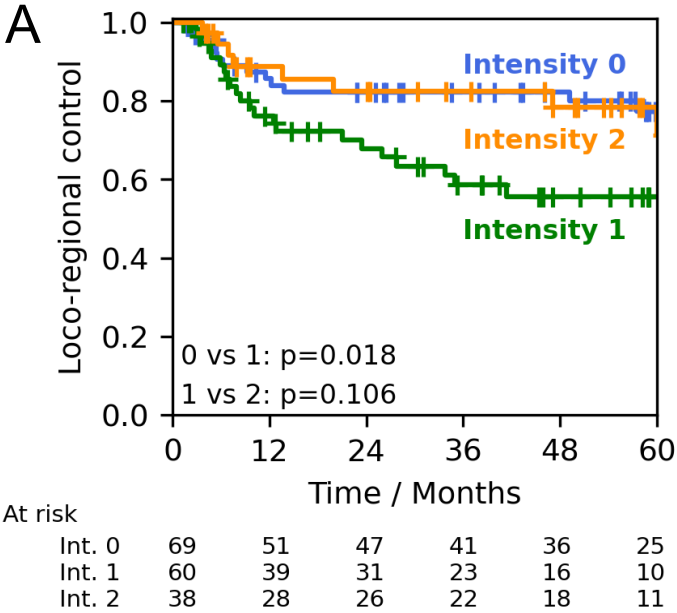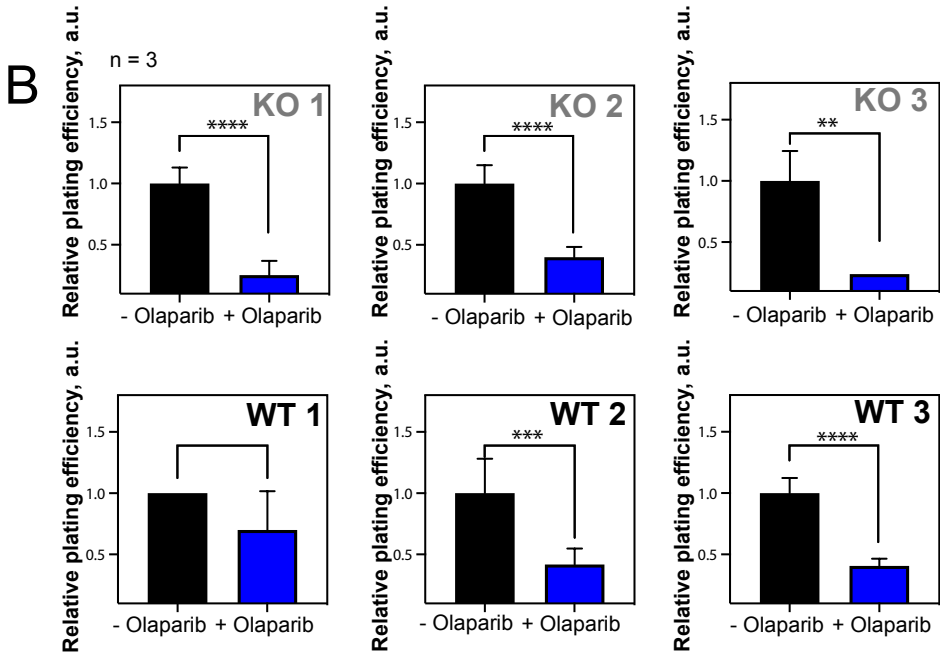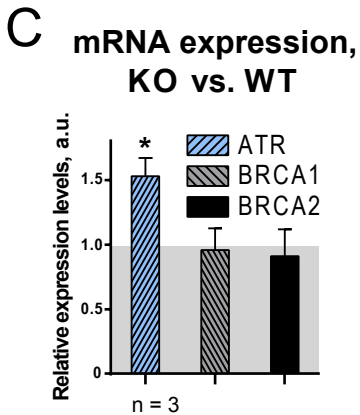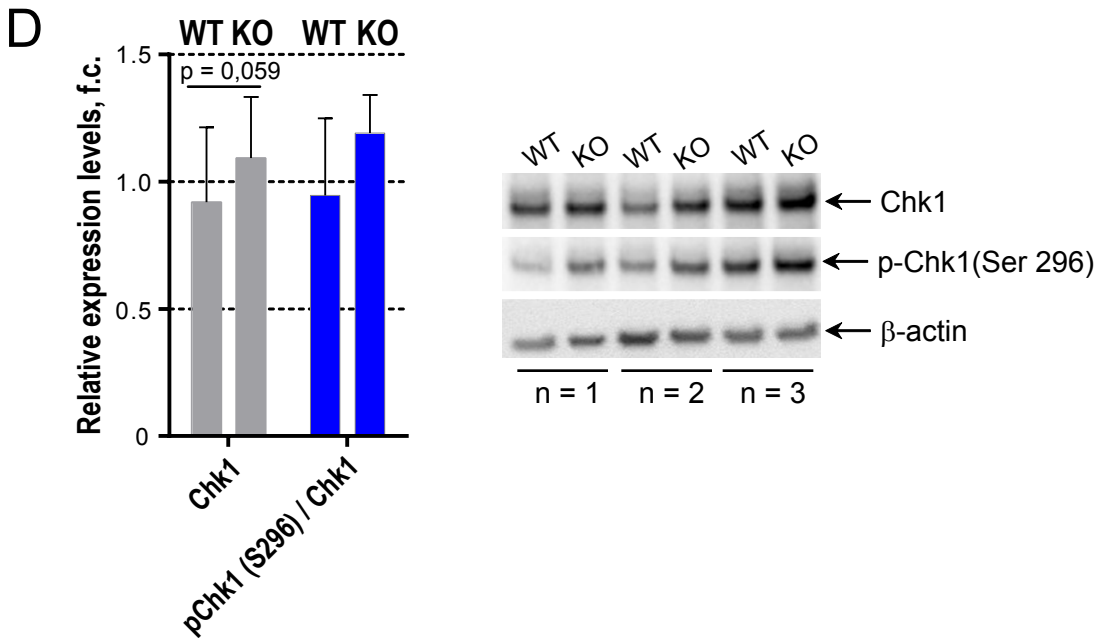

Supplement: Supplementary file 8 — Supplementary Figure 5 [file 41388_2021_1842_MOESM8_ESM.pdf]

Supplementary Figure 6

A

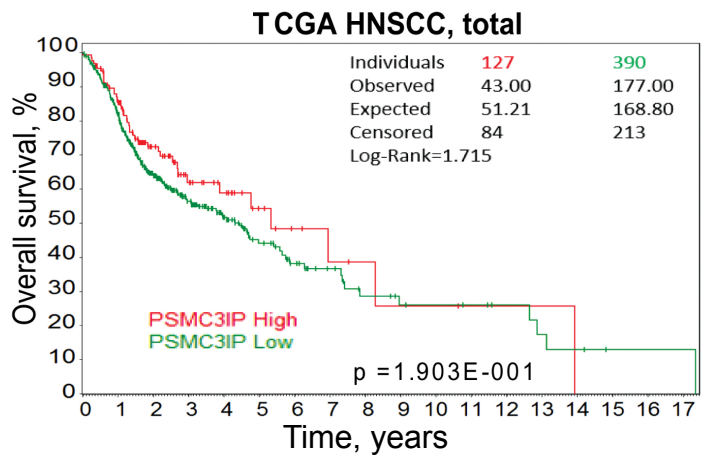

B

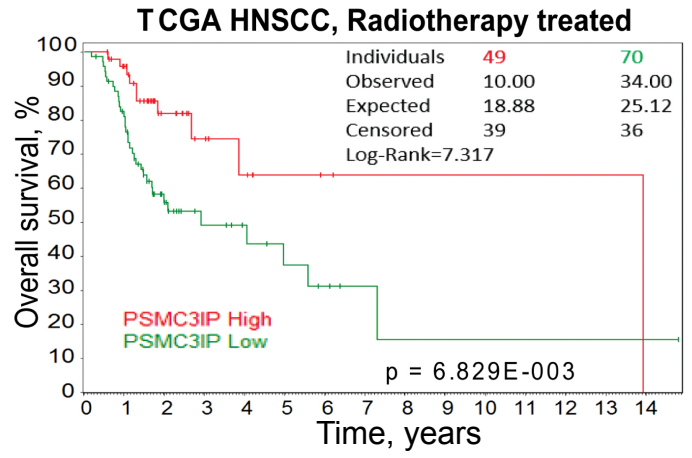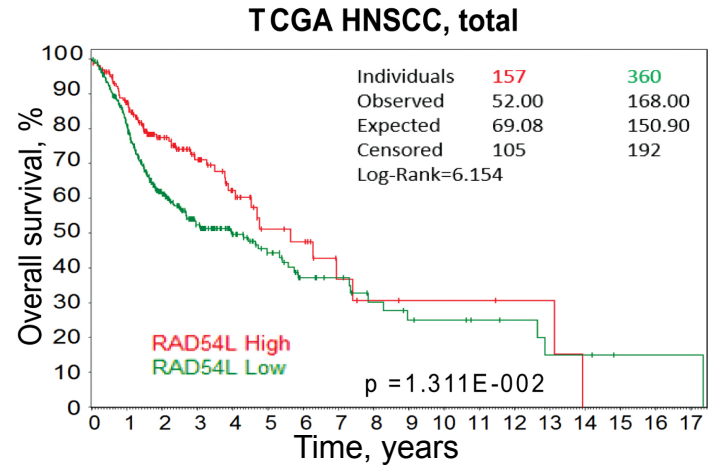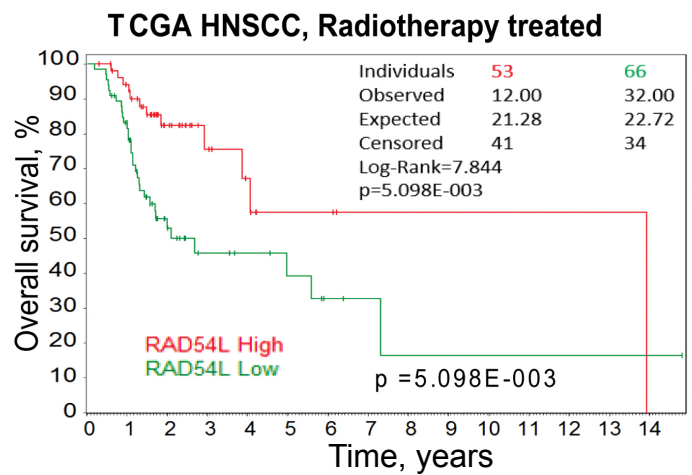

Supplement: Supplementary file 9 — Supplementary Figure 6 [file 41388_2021_1842_MOESM9_ESM.pdf]

Supplementary Figure 8

A

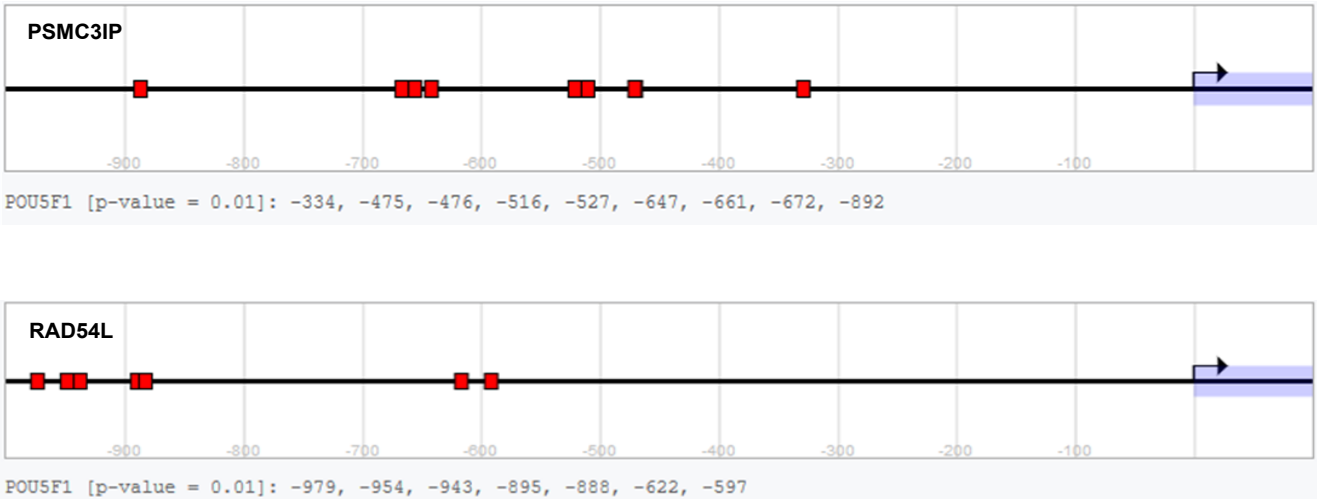

B

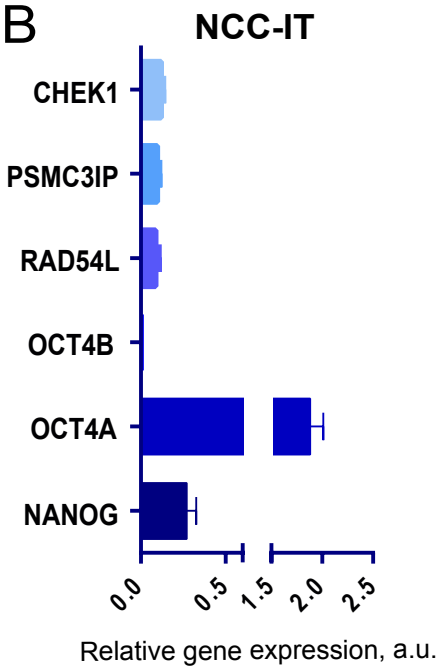

C

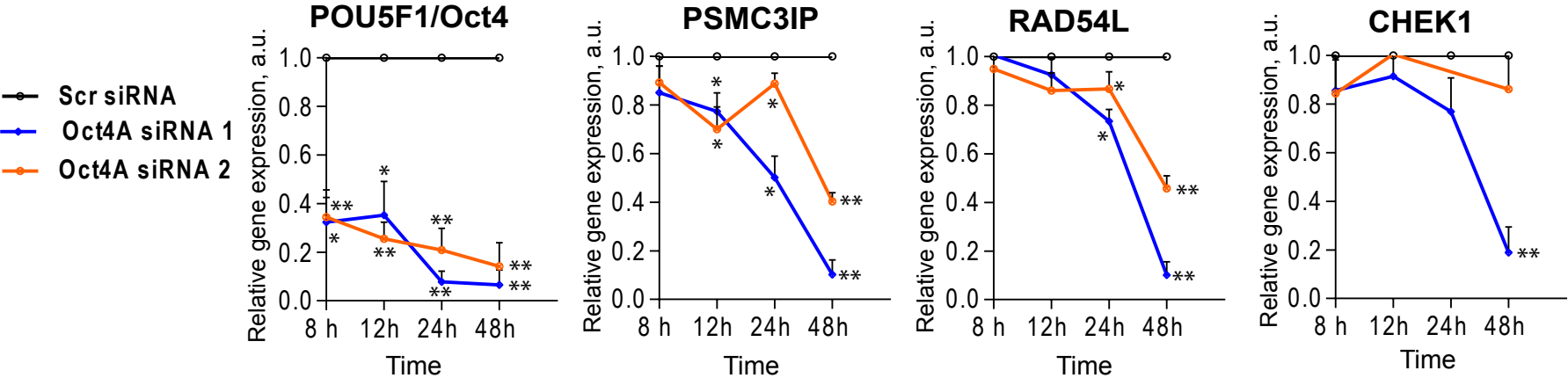

Supplement: Supplementary file 11 — Supplementary Figure 8 [file 41388_2021_1842_MOESM11_ESM.pdf]

Supplementary Figure 9

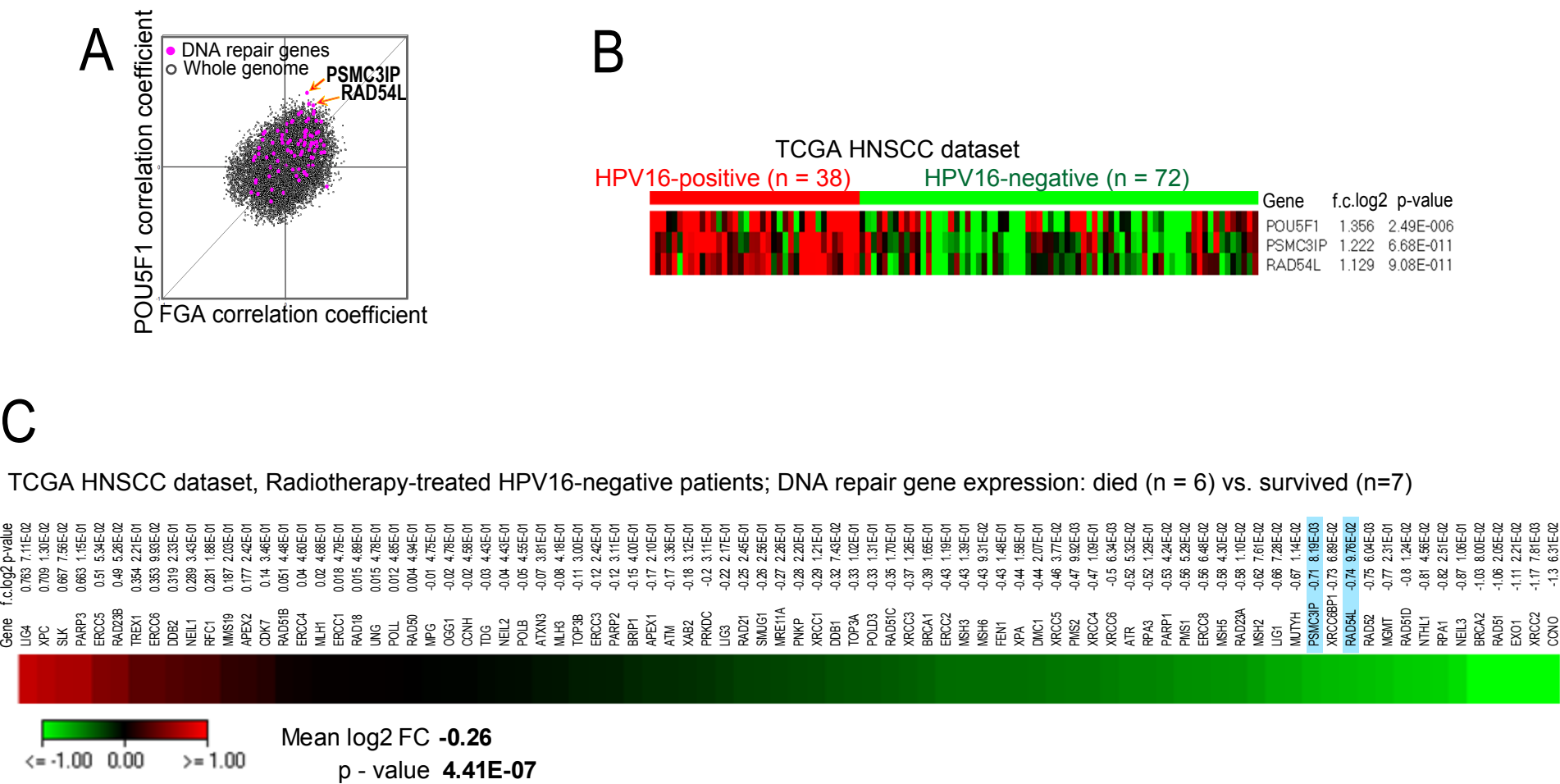

Supplement: Supplementary file 12 — Supplementary Figure 9 [file 41388_2021_1842_MOESM12_ESM.pdf]

Supplementary Figure 10

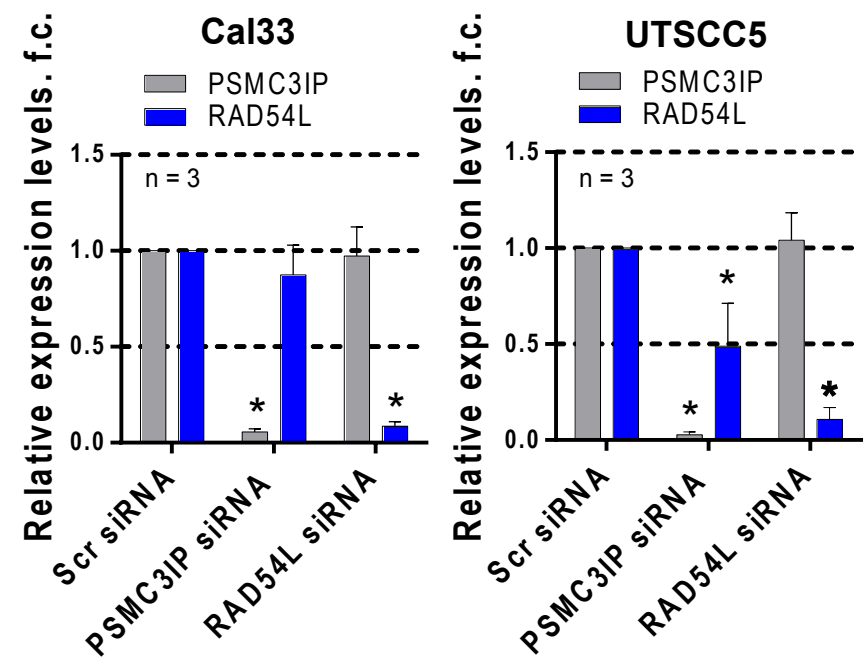

Supplement: Supplementary file 13 — Supplementary Figure 10 [file 41388_2021_1842_MOESM13_ESM.pdf]

Supplementary Figure 11

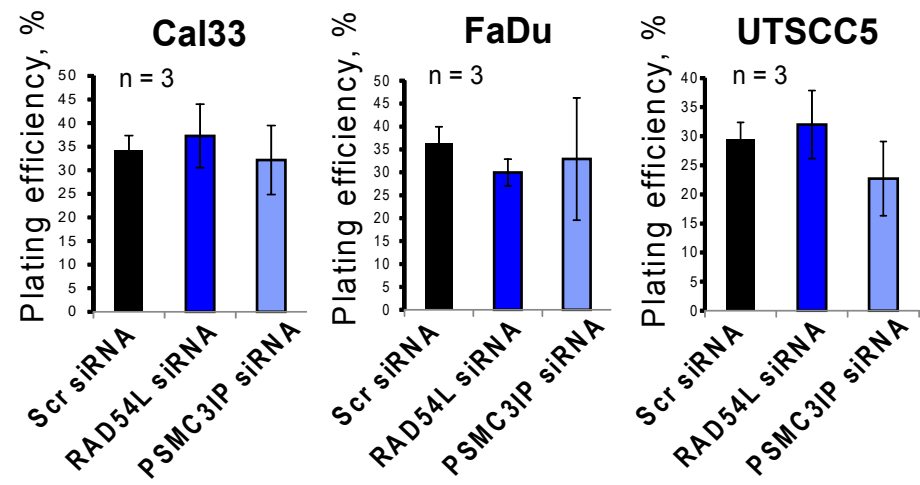

Supplement: Supplementary file 14 — Supplementary Figure 11 [file 41388_2021_1842_MOESM14_ESM.pdf]
