# Supplementary material for: Oct4 confers stemness and radioresistance to head and neck squamous cell carcinoma by regulating the homologous recombination factors PSMC3IP and RAD54L
Source: Oncogene. 2021 Jun 2;40(24):4214–28. doi: 10.1038/s41388-021-01842-1 (PMC8211562; doi:10.1038/s41388-021-01842-1)

Supplementary Figure 7

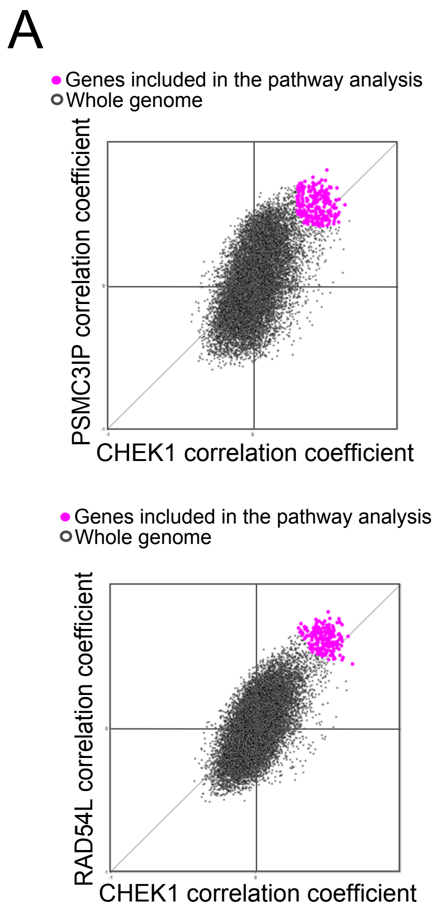

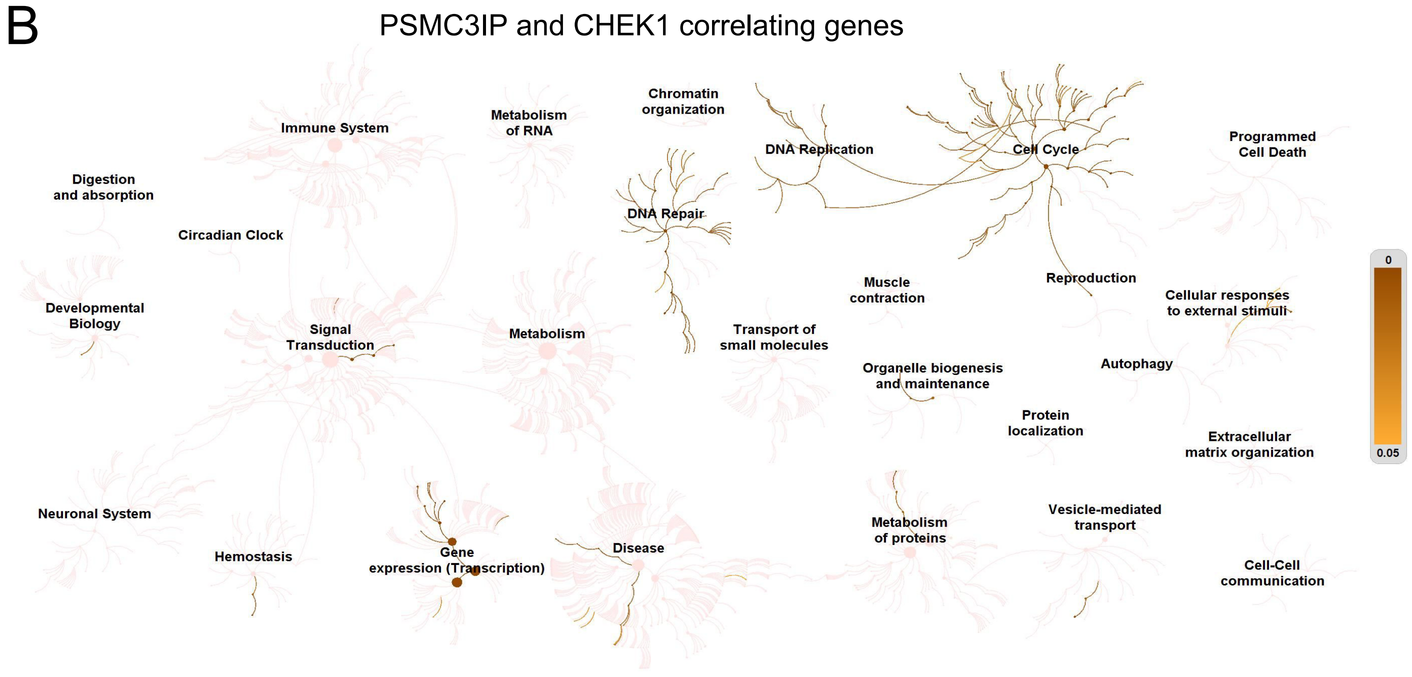

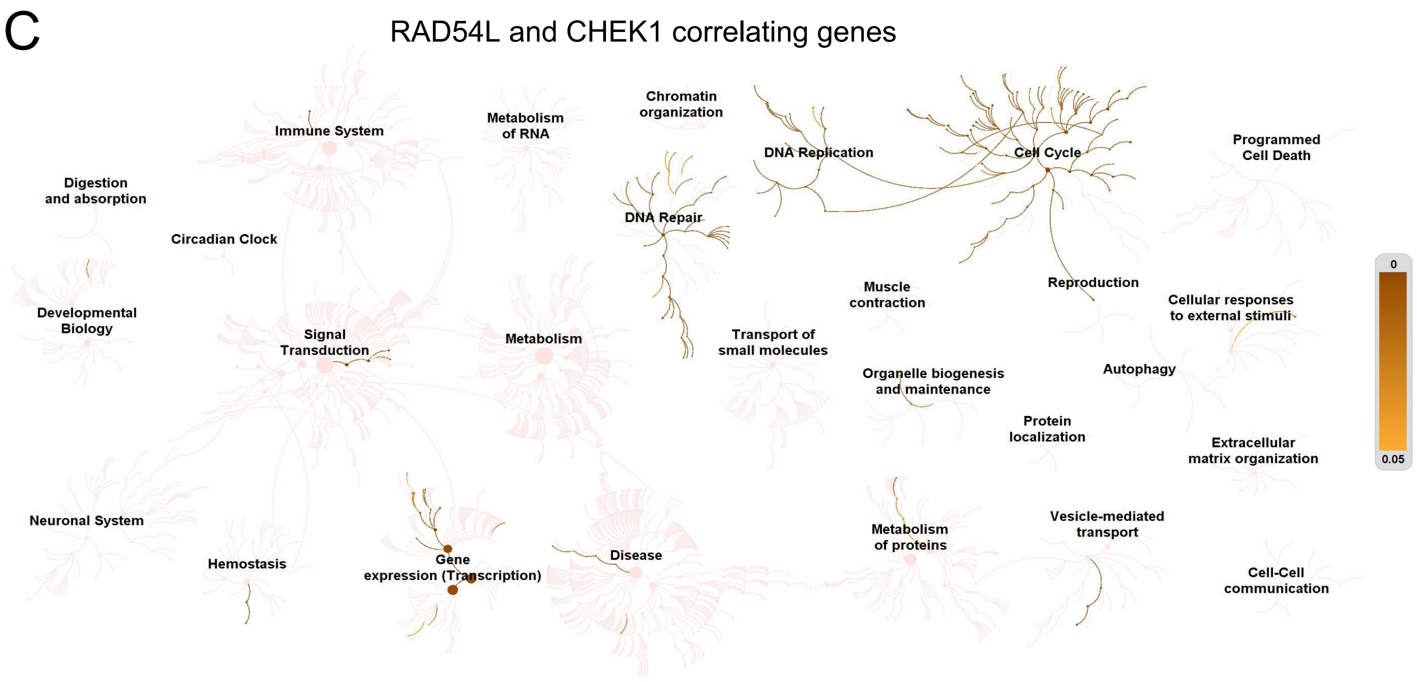

Supplement: Supplementary file 10 — Supplementary Figure 7 [file 41388_2021_1842_MOESM10_ESM.pdf]
